# Genomic consequences of population decline in critically endangered pangolins and their demographic histories

**DOI:** 10.1093/nsr/nwaa031

**Published:** 2020-02-27

**Authors:** Jing-Yang Hu, Zi-Qian Hao, Laurent Frantz, Shi-Fang Wu, Wu Chen, Yun-Fang Jiang, Hong Wu, Wei-Min Kuang, Haipeng Li, Ya-Ping Zhang, Li Yu

**Affiliations:** 1 State Key Laboratory for Conservation and Utilization of Bio-Resource in Yunnan, School of Life Sciences, Yunnan University, Kunming 650091, China; 2 State Key Laboratory of Genetic Resources and Evolution, Kunming Institute of Zoology, Chinese Academy of Sciences, Kunming 650223, China; 3 CAS Key Laboratory of Computational Biology, CAS-MPG Partner Institute for Computational Biology, Shanghai Institute of Nutrition and Health, Chinese Academy of Sciences, Shanghai 200031, China; 4 University of Chinese Academy of Sciences, Beijing 100049, China; 5 School of Biological and Chemical Sciences, Queen Mary University of London, London E1 4NS, UK; 6 The Palaeogenomics and Bio-Archaeology Research Network, Department of Archaeology, University of Oxford, Oxford OX1 3TG, UK; 7 Kunming College of Life Science, University of Chinese Academy of Sciences, Kunming 650204, China; 8 Guangzhou Zoo, Guangzhou 510070, China; 9 Lushui Management and Conservation Branch of Gaoligong Mountain National Nature Reserve, Nujiang 673100, China; 10 Center for Excellence in Animal Evolution and Genetics, Chinese Academy of Sciences, Kunming 650223, China

**Keywords:** pangolins, population genomics, population declines, genetic diversity, inbreeding, genetic load, demographic history

## Abstract

Pangolins are among the most critically endangered animals due to heavy poaching and worldwide trafficking. However, their demographic histories and the genomic consequences of their recent population declines remain unknown. We generated high-quality *de novo* reference genomes for critically endangered Malayan (*Manis javanica*, MJ) and Chinese (*M. pentadactyla*, MP) pangolins and re-sequencing population genomic data from 74 MJs and 23 MPs. We recovered the population identities of illegally traded pangolins and previously unrecognized genetic populations that should be protected as evolutionarily distinct conservation units. Demographic reconstruction suggested environmental changes have resulted in a population size fluctuation of pangolins. Additionally, recent population size declines due to human activities have resulted in an increase in inbreeding and genetic load. Deleterious mutations were enriched in genes related to cancer/diseases and cholesterol homeostasis, which may have increased their susceptibility to diseases and decreased their survival potential to adapt to environmental changes and high-cholesterol diets. This comprehensive study provides not only high-quality pangolin reference genomes, but also valuable information concerning the driving factors of long-term population size fluctuations and the genomic impact of recent population size declines due to human activities, which is essential for pangolin conservation management and global action planning.

## INTRODUCTION

Pangolins (monotypic order Pholidota and family Manidae) are believed to be the most heavily poached and trafficked mammals in the world, accounting for as much as 20% of all illegal transnational wildlife trade [1–4]. Overexploitation driven by escalating demand for their meat as luxury food consumption and scales for traditional medicines has driven pangolins to the edge of extinction [5–10]. More than 1 million pangolins had been poached in the decade prior to 2014 [11]. All eight pangolin species (four Asian pangolins: Malayan pangolin *Manis javanica*, Chinese pangolin *M. pentadactyla*, Indian pangolin *M. crassicaudata* and Philippine pangolin *M. culionensis*; four African pangolins: Tree pangolin *Phataginus tricuspis*, Long-tailed pangolin *P. tetradactyla*, Temminck's ground pangolin *Smutsia temminckii* and Giant pangolin *S. gigantean*) [12,13] have been listed in ‘Appendix I’ of the Convention on International Trade in Endangered Species of Wild Fauna and Flora (CITES) [14]. Notably, two Asian pangolins, Malayan and Chinese pangolins, are the most threatened pangolin species with the highest extinction risk. The Malayan pangolin is mainly found in Southeast Asia [9,14,15] and the Chinese pangolin mainly inhabits the south of China and some of northern Southeast Asia [16–18]. Both species have been recognized as the most-traded mammals in Asia [2,5,7,14] and classified as Critically Endangered on the International Union for Conservation of Nature (IUCN) red list [19]. Although a zero-trade quota for wild-caught individuals of them has been exerted since 2000 [20], wildlife poaching and international illegal trades have been still reported and the population decline continues [2,5,9,14].

The two critically endangered pangolins have attracted much attention. Earlier studies have focused on the confiscated species identification [6,9,13,21] and the phylogeny [12,22,23] by using mitochondrial (mt) genes and microsatellites. Recent genomic studies have investigated the illegal trade routes [15] and the adaptive evolution of pangolin scales and immunity [16], while several transcriptomic studies have explored the adaptive evolution of their myrmecophagous feeding [24,25]. However, very limited knowledge was available about their demographic histories and the genomic consequences associated with their recent population declines, which is essential for pangolin conservation management and global action planning.

Therefore, in this study, we have generated a data set comprising two high-quality *de novo* assembled genomes of Malayan and Chinese pangolins, which are significantly improved compared with the published pangolin assemblies [16,26], and re-sequenced the genomes of 74 Malayan and 23 Chinese pangolins. The population genomic analyses provide a comprehensive evaluation of their survival potential, the driving factors of long-term population size fluctuations and the genomic impact of recent population size declines due to human activities.

## RESULTS AND DISCUSSION

### Generation of two high-quality pangolin reference genomes

The two newly assembled pangolin genomes were generated with a very-high-coverage whole-genome shotgun and 10X genomic sequencing strategy. We generated a total of 980 Gb of sequence data (411.8-fold genome coverage) from a Malayan pangolin (*M. javanica*, referred to hereafter as MJ) and 831 Gb of sequence data (289.6-fold genome coverage) from a Chinese pangolin (*M. pentadactyla*, referred to hereafter as MP) (Supplementary Table 1).

The genome sizes of the *de novo* assembly for MJ and MP are 2.45 and 2.40 Gb, with scaffold N50 of 13.85 and 7.77 Mb, respectively (Supplementary Table 2). The base error rates for the reference genomes are $5.0 \times {10^{ - 6}}$ and $4.5 \times {10^{ - 6}}$ for MJ and MP, respectively, which are both <$1.0 \times {10^{ - 5}}$. Both of them are significantly improved compared with the published pangolin assemblies (genome size: 2.55 and 2.21 Gb, scaffold N50: 0.2 and 0.16 Mb in Choo *et al.* [16]; genome size: 2.85 and 2.91 Gb, scaffold N50: 1.97 and 1.88 Mb in Huang *et al.* [26]). For the MJ genome, 94.93% of the short reads were mapped onto the 99.98% assembled genome and, for the MP genome, 98.20% of the short reads were mapped onto the 99.96% assembled genome, indicating high reliability of both pangolin genome assemblies.

To annotate the genomes, we combined three approaches to predict genes including *ab initio* gene prediction, RNA-seq data and homology-based gene prediction. We identified 21 785 and 23 865 protein-coding genes in the Malayan and Chinese pangolin genomes, respectively. The numbers of identified protein-coding genes are similar to those in the previous study (23 446 and 20 298 for the two pangolins) [16]. Core Eukaryotic Genes Mapping Approach (CEGMA) [27] evaluation of MJ and MP genome assemblies indicated that 91.13% and 89.52% of 248 core eukaryotic genes are complete. However, in the published pangolin assemblies [16], only 58% and 55% of 248 core eukaryotic genes are complete in the CEGMA evaluation. Benchmarking Universal Single-Copy Orthologs (BUSCO) [28] assessment of the two new pangolin assemblies also showed that 94.10% and 94.50% of 4104 single-copy mammalian orthologous genes are complete (Supplementary Table 2). Thus, the new genome assemblies provide better coverage for protein-coding genes than the previous ones [16].

In summary, the two newly assembled pangolin genomes were both significantly improved compared with the previous pangolin genome assemblies [16,26] (Supplementary Table S2) and the high-quality pangolin reference genomes are not only important for the population genomic analyses of the pangolin, but also essential for mammalian study.

### Generation of population genomic data and variant discovery

Individual genome re-sequencing of 74 MJs at an average 15.15-fold coverage (ranging between 12.12 and 21.16 for different individuals) and 23 MPs at an average of 38.64-fold coverage (ranging between 31.92 and 45.51 for different individuals) were generated for population genomic analyses (Supplementary Table 3). The average alignment rate to the reference genomes are 96.75% (88.71%–98.42%) and 98.80% (97.98%–99.44%) for Malayan and Chinese pangolins, respectively (Supplementary Table S3). The high alignment rate ensures the accurate identification of genetic variation. The single-nucleotide polymorphism (SNP) calling and filtering produced a total of 20.02 and 21.82 million high-quality autosomal SNPs for MJs and MPs, respectively.

### Revealing population identity for illegally traded pangolins

**Figure 1.**
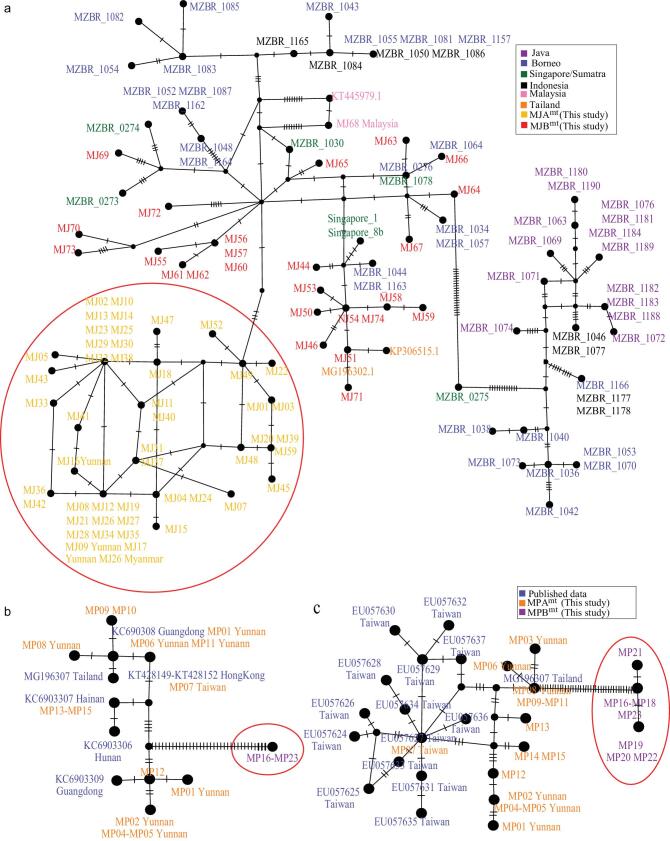
Haplotype network analyses based on the extracted mt-gene sequences of our samples and those of previously available samples with known origin. The newly defined MJA^mt^ and MPB^mt^ populations in this study are framed in a red circle. (a) The concatenated *CYTB* and *COI* gene sequences from 74 Malayan pangolin individuals (MJ01–74) in this study and those of 60 individuals in previous studies. Purple : Java [15], blue : Borneo [15], green : Singapore/Sumatra [15], black : Indonesia [15], orange : Thailand [12,23], pink : Malaysia (NCBI GenBank), yellow : MJA^mt^ (this study; all are framed in a red circle; samples of known geographic locations are labeled with location names after the sample code names), red : MJB^mt^ (this study; samples of known geographic locations are labeled with location names after the sample code names). (b) The *COI* gene sequences from 23 Chinese pangolin individuals (MP01–23) in this study and those of 10 individuals in previous studies [9,12]. (c) The *CYTB* gene sequences from 23 Chinese pangolin individuals (MP01–23) in this study and those of 15 individuals in previous studies [6,12]. Blue : published data; orange : MPA^mt^ (this study), purple : MPB^mt^ (this study; all are framed in a red circle). Samples of known geographic locations are labeled with location names after the sample code names.

**Figure 2.**
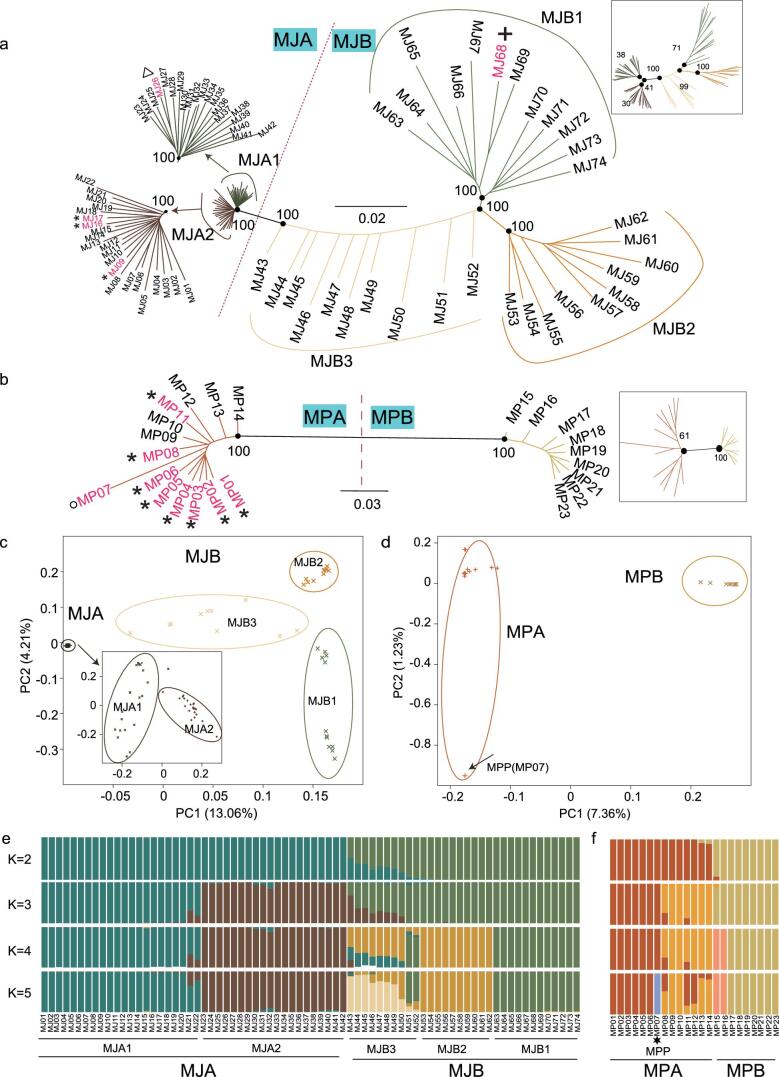
Phylogenetic analyses, PCA and admixture analyses based on nuclear genome sequences. (a) NJ phylogeny of 74 Malayan pangolins (MJ) (5 samples with known geographic sources; * Yunnan, ${\vartriangle}$ Myanmar, + Malaysia). ML phylogeny produced similar results and is shown as a small version to the right of the figure as a sub-figure. Two populations (MJA and MJB) and subpopulations within the two populations (MJA1-2 and MJB1-3) are labeled. MJA1 and MJA2 are also shown as a magnified version in the top-left of the figure. The values around the black point represent bootstrap support. (b) NJ phylogeny of 23 Chinese pangolins (MP) (9 samples with known geographic sources; * Yunnan, o Taiwan island). ML phylogeny produced similar results and is shown as a small version to the right of the figure as a sub-figure. Two populations (MPA and MPB) are labeled. The values around the black point represent bootstrap support. (c) Principal component analysis (PCA) for 74 MJ individuals. PCA for the MJA population is also shown. (d) PCA for 23 MP individuals. (e) Admixture analyses for 74 MJ individuals. The postulated number of ancestral clusters (K) was set from 2 to 5. (f) Admixture analyses for 23 MP individuals. The postulated number of ancestral clusters (K) was set from 2 to 5.

We traced the origin locations of illegally traded pangolins in this study, which has been collected from different times and different places, via genetic clustering with pangolins of known origin. The most available molecular data of pangolins are mt DNA data. In this study, deep coverage of the mt genome allowed us to obtain the accurate mt sequences for all pangolin samples. The mt data of these Malayan pangolins were combined together with the publicly available mt data of Malayan pangolins from multiple Southeast Asian countries, including Indonesia (Borneo, Java and Sumatra), Singapore, Thailand and Malaysia [12,15,23]. The haplotype network analyses revealed two clusters of Malayan pangolins (Fig. 1a), named MJA^mt^ and MJB^mt^ populations. These two clusters were also distinguished by the nuclear genome tree of the Malayan pangolin, named MJA and MJB populations (Fig. 2a). The MJA^mt^ and MJA populations contained the individuals sampled from the mainland (MJ09, MJ16 and MJ17 from Yunnan and MJ26 from Myanmar) that were divergent from other samples, corroborating the MJA’s non-overlapping geographic locality that stands for the mainland population. In comparison, the MJB^mt^ and MJB populations contained the individuals sampled from the Southeast Asian islands, except for Java (Fig. 1a). The relatively deep splits among MJB lineages (MJB1–3; Fig. 2a) are also expected because of the isolation effect of islands.

The results of principal component analysis (PCA) (Fig. 2c) and the admixture analysis (Fig. 2e) from the nuclear genomes recapitulated the phylogenetic and network analysis results in Malayan pangolins. The first PCA component (PC1) separates MJA and MJB (Tracy-Widom, *P* = ${\rm{1}}{\rm{.57\ \times }}\ {\rm{1}}{{\rm{0}}^{- {\rm{21}}}}$) and the second component (PC2) divides MJB into subpopulations (MJB1–3; *P* = 7.28 ${\rm{ \times }}$ 10^−4^) (Fig. 2c). Moreover, the PCA also revealed two subpopulations within MJA (MJA1 and MJA2; *P* = 3.15 ${\rm{ \times }}$ 10^−6^) (Fig. 2c). The admixture analysis (Fig. 2e) showed the lowest cross-validation error when K = 2 (Supplementary Fig. 1) and supported two genetically distinct populations in the Malayan pangolins. When K = 5, MJs were separated into five clusters (Fig. 2e), corresponding to five subpopulations identified in the phylogenetic analyses and PCA.

Based on mt DNA, the haplotype network analyses revealed two clusters of Chinese pangolins (Figs. 1b and c), named MPA^mt^ and MPB^mt^ populations. The MPA^mt^ population was composed of the samples that originated from Yunnan, Guangdong, Hunan, Hainan, Taiwan in China and Thailand [6,9,12]. Interestingly, the MPB^mt^ population appeared as an independent cluster, which is highly diverged from the MPA^mt^ population and has short intra-population branches. All of the MPB individuals were seized from at least four illegal trades on the Sino–Burmese border between 2016 and 2017 (Supplementary Table 3). Therefore, it is very likely that MPB individuals originate from Myanmar. We suggest that the MPB population needs conservation attention for their unique evolutionary status. The same two populations (MPA and MPB) were inferred from the nuclear genome tree (Fig. 2b). Similarly to the network inferred from the mt DNA, the intra-population branches of the MPB population in the nuclear genome tree are short. Interestingly, within the MPA population, the individual from Taiwan [17] (number MP07; the representative of the Taiwan subspecies *M. pentadactyla pentadactyla* in the previous study [16], referred to hereafter as MPP) demonstrated a significantly long branch compared with the other MPA individuals (Wilcox-test; *P *< 0.05 in neighbor-joining (NJ) and maximum-likelihood (ML) trees). In the PCA results, PC1 separated MPA and MPB (*P* = 3.46 ${\rm{ \times }}$ 10^−4^) (Fig. 2d). PC2 divided Taiwan pangolins from other MPA pangolins, although it was marginally significant (*P* = 0.055). The admixture analysis (Fig. 2e) showed the lowest cross-validation error when K = 2 (Supplementary Fig. 1) and supported two genetically distinct populations in the Chinese pangolins. For the Taiwan pangolin individual, it showed an MPA genetic component from K = 2 to 4 (Fig. 2f). At K = 5, a component specific to the Taiwan individual then appeared.

Intriguingly, for both pangolin species, some individuals demonstrated the signal of admixture between two populations at K = 2 (Fig. 2e and f). Particularly, all individuals of the MJB3 subgroup were primarily formed by admixture of the ancestral MJA and MJB populations (Fig. 2e). The admixture between the populations within MJs and MPs was also reflected by the finding that these admixed individuals varied between the mt haplotype network and the nuclear genome tree due to migration (see Figs 1 and 2).

As a summary, we combined the mt and nuclear genome data together to trace the population identity of the illegally traded pangolins. MJA pangolins are continentally distributed, while MJB pangolins are from the Southeast Asian islands, except

Java. The MPA pangolins are composed of the samples that originated from southern China and Thailand, while the MPB pangolins are most likely to originate from Myanmar.

### Genetic diversity of pangolins

**Figure 3.**
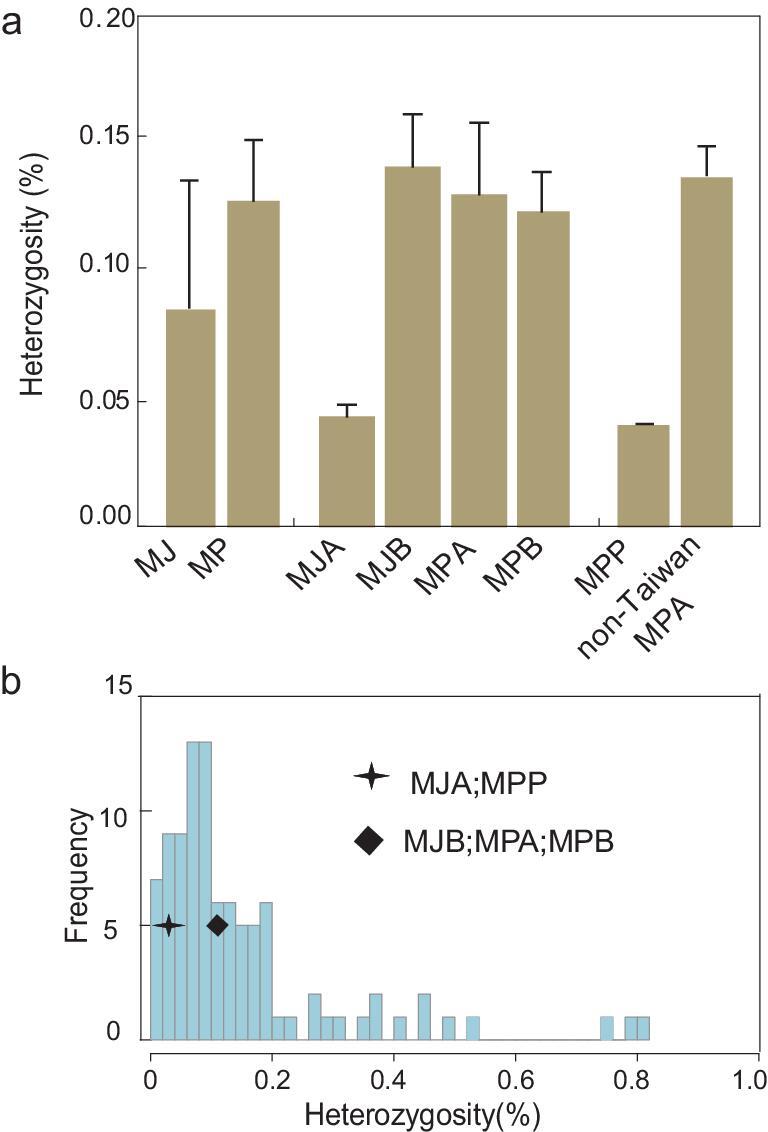
Genetic diversity of pangolins. Error bars represent SD. (a) Heterozygosity of MJ, MP, MJA, MJB, MPA, MPB, MPP and non-Taiwan MPA. (b) The frequency of the distribution of the genome-wide heterozygosity in 94 published mammals (Supplementary Table 4) and pangolins.

The mean heterozygosity (*H_e_*) of MP (0.127%) is significantly higher than that of MJ (0.085%) (Wilcox-test, *P* = 4.79 ${\rm{ \times }}$ 10^−3^; Fig. 3a). Among the four main populations, the mean heterozygosity of MJA (${H_e} = \ $0.043%) is the lowest compared with the other three main populations (MJB, MPA, MPB) (${H_e} = \ $0.141%, 0.130% and 0.123%, respectively; *P* = 2.34 ${\rm{ \times }}$ 10^−13^, 8.20 ${\rm{ \times }}$ 10^−7^ and 3.18 ${\rm{ \times }}$ 10^−6^; Fig. 3a). These observations suggest that the significant difference in ${H_e}$ between MJ and MP is mainly due to the severely reduced genetic diversity of MJA.

The ${H_e}$ of the Taiwan pangolin (MPP) (0.04%) is notably lower than the ${H_e}$ of the other MPA pangolins (0.125%–0.162%; *P* = $8.17{\rm{\ }} \times {10^{ - 4}}$), MPB pangolins (0.107%–0.155%; *P* = $4.55{\rm{\ }} \times {10^{ - 3}}$) and MJB pangolins (0.11%–0.17%; *P* = $4.16{\rm{\ }} \times {10^{ - 7}}$), but similar to the extremely low ${H_e}$ of MJA pangolins (0.036%–0.062%) as described above (Fig. 3a). The low genetic diversity in MPP is most likely due to the small founding population on the island. Moreover, both phylogenetic and PCA analyses (Fig. 2b and d) indicate that MPP is indeed a differentiated and isolated population.

Next, we compared the ${H_e}$ of pangolins with that of other species (Fig. 3b and Supplementary Table 4). The ${H_e}$ of MJA is similar to those of the endangered golden snub-nosed monkeys (0.042%) [29] and carnivores, including the Bengal tiger, White lion, Amur tiger and San Nicolis Island fox (0.040%–0.049%) [30–32], whereas those of MJB, MPA and MPB were comparable to those in the endangered Siamang (0.13%–0.15%) [33], giant panda (0.132%) [34], critically endangered Sumatran orangutan (0.12%) [35], Sumatran Rhinoceros (0.13%) [36], Western lowland gorilla (0.144%) [37] and the extinct Oimyakon woolly mammoth (0.125%) [38]. Such low level of genetic diversity

suggests that pangolins likely have decreased adaptive potential.

### Demographic history of pangolins

**Figure 4.**
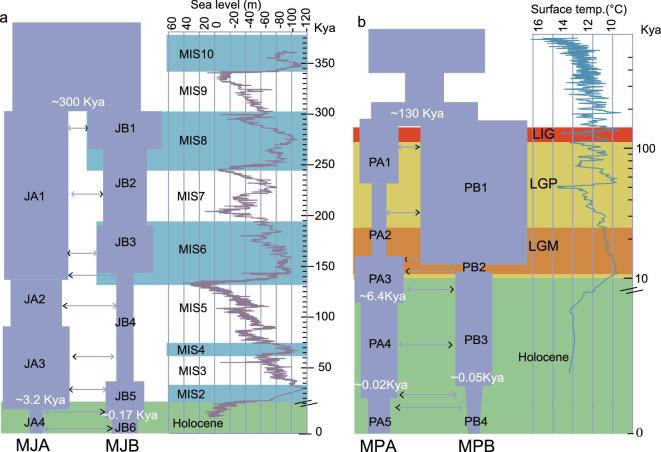
Demographic histories of MJ (a) and MP (b) inferred from the demographic model simulations. The arrows indicate migration events and those with stronger migration events in pairwise comparisons are indicated with bold arrows ((a) and (b)). The blue shadows represent the marine isotope stage (MIS) during the Pleistocene [40] (a). The red and orange shadows represent the last interglacial period and last glacial period (LGP) during the Pleistocene, respectively (a). The green shadows represent the Holocene era ((a) and (b)). The surface temperature (temp.) over the past 1 million years based on δ^18^O data [42] is indicated (b).

The ancient demography of pangolins is very important for understanding the driving factors, i.e. the environmental changes, behind the population fluctuation of pangolins in the past. The joint site-frequency spectrum (joint SFS) is one of the optimal summary statistics for addressing these issues and the parameters can be reliably inferred by coalescent-based simulations [39]. Thus, we assessed various demographic scenarios of MJ and MP species by coalescent-based simulations (Supplementary Figs 2 and 3 and Supplementary Tables 5–7). Based on the best-fitting model, the parameters, including divergence time, current effective population size and migration rate, were estimated (Supplementary Tables 8–10). All these pangolin populations were subject to multiple population size changes in their evolutionary history (Fig. 4).

Two Malayan-pangolin populations (MJA and MJB) split 300 thousand years ago (Kya), corresponding to the beginning of marine isotope stage (MIS) 8 [40]—a period during which the sea level fell precipitously, potentially resulting in land-habitat expansion and climate changes, and contributing to the population differentiation. Interestingly, the MJA population showed a small population size fluctuation until a severe population size decline at 3.2 Kya (from about 106 000 to 2000 individuals; Supplementary Table 8). The MJB population mainly lives on islands and demonstrates numerous changes in population size, potentially as a result of sea-level changes (Fig. 4a). The subsequent MIS periods associated with alternating episodes of sea-level fall (i.e. MIS8, MIS6 and MIS2) and rise (i.e. MIS7 and MIS5e) [40–42] partially explained the population expansions and bottlenecks (Fig. 4a), respectively. The close conjunction of population size fluctuation and the sea-level change accords with known and potential locality sources of MJB samples from Southeast Asian islands (Fig. 1a).

Two Chinese pangolin populations (MPA and MPB) split at 130 Kya when the last interglacial period began [43] and the most recent and intense uplift event of the Tibetan plateau occurred (i.e. the Gonghe movement) [44]. These two factors were known to have led to the speciation of a large number of organisms in southern China [45]. Therefore, this indicates that the climate warming and the Tibetan-plateau uplift may also have contributed to the split of MP populations that mainly live in southern China [12,16,18]. After the split, MPA and MPB experienced an abrupt population decline around the last glacial period (LGP, ∼10–120 Kya) (70% reduction for MPA, from about 2300 to 12 000 individuals; corresponding to PA1 in Fig. 4b) or the last glacial maximum (LGM; ∼25–13 Kya [46]; 97% reduction for MPB, from about 110 000 to 2900 individuals; corresponding to PB2 in Fig. 4b) (Supplementary Table 8). Therefore, the impacts of LGP and LGM on the population-size decline were revealed for MP species. The similar phenomenon was also observed in the giant panda [47] and the golden snub-nosed monkeys [29,48], which geographically largely overlap with MP species.

**Figure 5.**
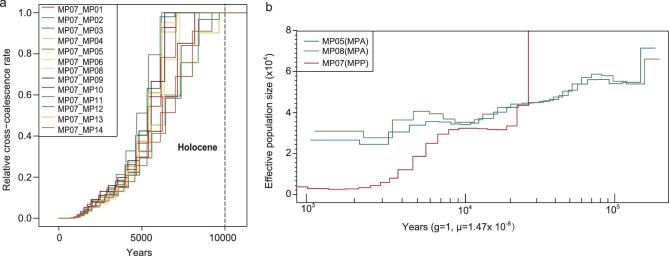
Demographic history of the Taiwan pangolin. (a) Divergence time of the Taiwan pangolin (MP07) from its ancestral MPA group (MP01–MP14). (b) Population size dynamics of the Taiwan pangolin (MP07) and non-Taiwan MPA. Two non-Taiwan MPA individuals (MP05 and MP08) that have the highest sequence coverage were used to represent the non-Taiwan MPA populations.

As a sub-species of Chinese pangolins, the Taiwan pangolin is of specific interest as an island-endemic pangolin. Based on phylogenetic and admixture analysis, the Taiwan pangolin was phylogenetically mixed with other MPA individuals and showed 100% MPA ancestry component (Fig. 2b and f), indicating that the Taiwan pangolin originated from the mainland counterpart MPA. Divergence time estimation (Fig. 5a) and demographic history reconstruction (Fig. 5b) showed that the Taiwan pangolin began to separate from its ancestral MPA group about 10 Kya, consistently with the fossil records documented for the immigration of numerous mammals, including the elephant and rhinoceros, from the mainland to Taiwan through a land bridge during the late Pleistocene (26–11 Kya) [49,50]. Afterwards, the Taiwan pangolin population went through a continuous and steep population decline.

### Effects of recent population decline on the inbreeding level and genetic load

The recent population decline due to human activities has been documented widely in pangolins [1,5,11,19]. We then studied the effects of the recent population decline on inbreeding levels by using the runs of homozygosity (ROH). A long ROH is an indicator of inbreeding arising within tens of generations [51,52], as reported in wolfs [51] and gorillas [52]. The long ROHs between MJ and MP indicate that the recent inbreeding occurred in pangolins <20 generations/years ago (Supplementary Fig. 4).

**Figure 6.**
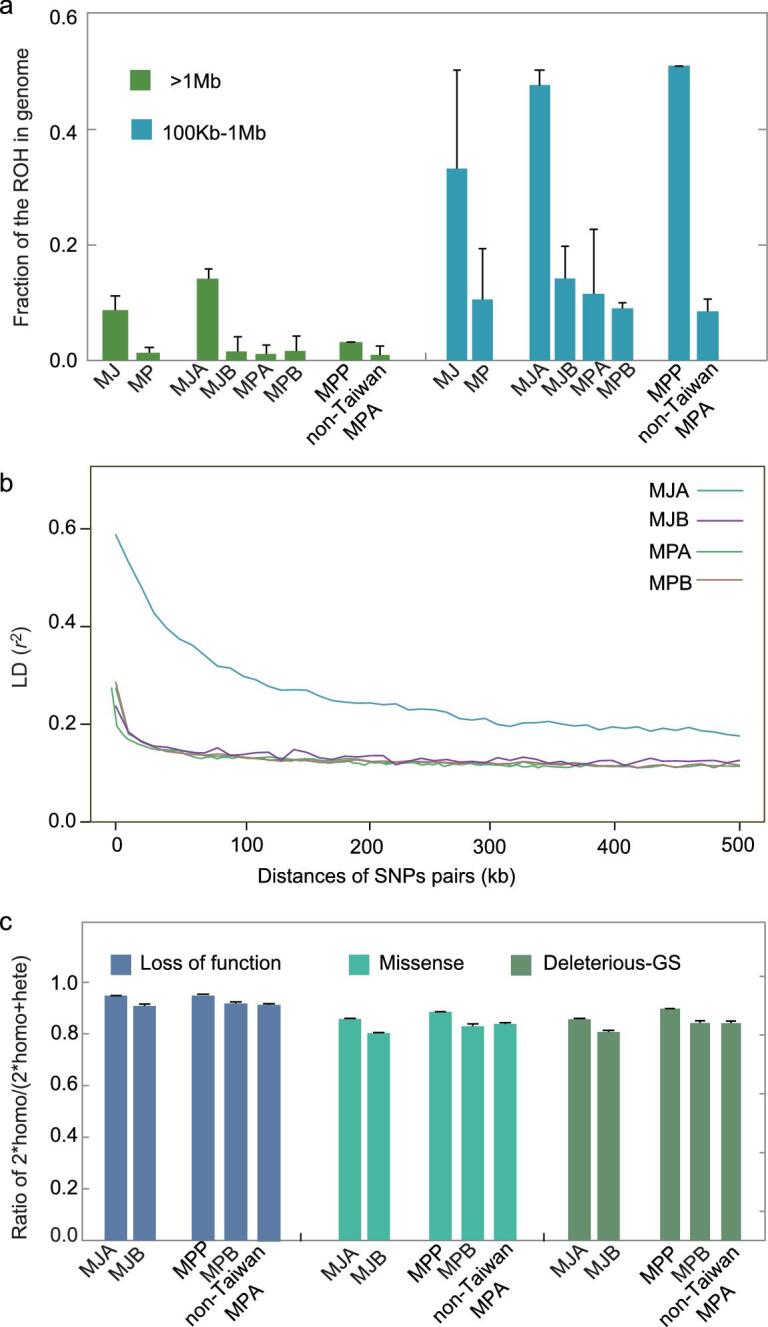
Inbreeding levels and genetic load of pangolins. Error bars represent SD. (a) Fraction of the runs of homozygosity (ROH) in the genome, including the medium (100 kb–1 Mb) and long (>1 Mb) ROH, of MJ, MP, MJA, MJB, MPA, MPB, MPP and non-Taiwan MPA. (b) Linkage disequilibrium (LD) of MJA, MJB, MPA and MPB. (c) Genetic load of MJA, MJB, MPP, non-Taiwan MPA and MPB.

Additionally, MJ pangolins harbored more ROHs >100 kb (974.26 Mb in total, 42.23% of the genome) than MP genomes (294.31 Mb in total, 11.99% of the genome) (Fig. 6a) and had significantly more long ROHs above 1 Mb (19.54 Mb in total, 8.8% of the genome) than those of MPs (3.54 Mb in total, 1.4% of the genome) (Wilcox-test, *P* = 1.34 ${\rm{ \times }}$ 10^–8^). Further comparisons among populations showed that MJA had more ROHs >100 kb (1559 Mb in total, 62.29% of the genome) than the other three populations (MJB, 390 Mb in total, 15.9% of the genome; MPA, 320 Mb in total, 12.79% of the genome; MPB, 269 Mb in total, 10.76% of the genome; Fig. 6a). MJA also had 8- to 12-fold more long ROHs above 1 Mb (34.91 Mb in total, 14.25% of the genome) than the other three populations (4.17, 2.89 and 4.18 Mb in total; 1.59%, 1.16% and 1.67% of the genome for MJB, MPA and MPB, respectively). In addition, consistently with the ROH results above, we found a longer extent of linkage disequilibrium (LD) in MJA than in the other three populations (Fig. 6b), which showed that LD decayed to *r*^2^ < 0.2 within 10 kb in MJB, MPA and MPB, but declined much more slowly for MJA (>300 kb). Overall, these results suggested the substantial increase of recent inbreeding in the MJ species, especially in the MJA population, possibly due to the recent human impact in recent decades.

We also assessed the inbreeding extent in the Taiwan pangolin. ROH analyses showed that the Taiwan pangolin population had more ROHs >100 kb (1366 Mb in total, 54.65% of the genome) than its mainland counterpart MPA (239 Mb in total, 9.57% of the genome) and MPB populations (269 Mb in total, 10.76% of the genome) (Fig. 6a). It also had more long ROHs above 1 Mb (8.04 Mb in total, 3.22% of the genome) than the latter two (2.49 Mb in total, 1.00% of the genome; 4.18 Mb in total, 1.67% of the genome). These results demonstrated that the Taiwan population also experienced an increase in recent inbreeding.

**Figure 7.**
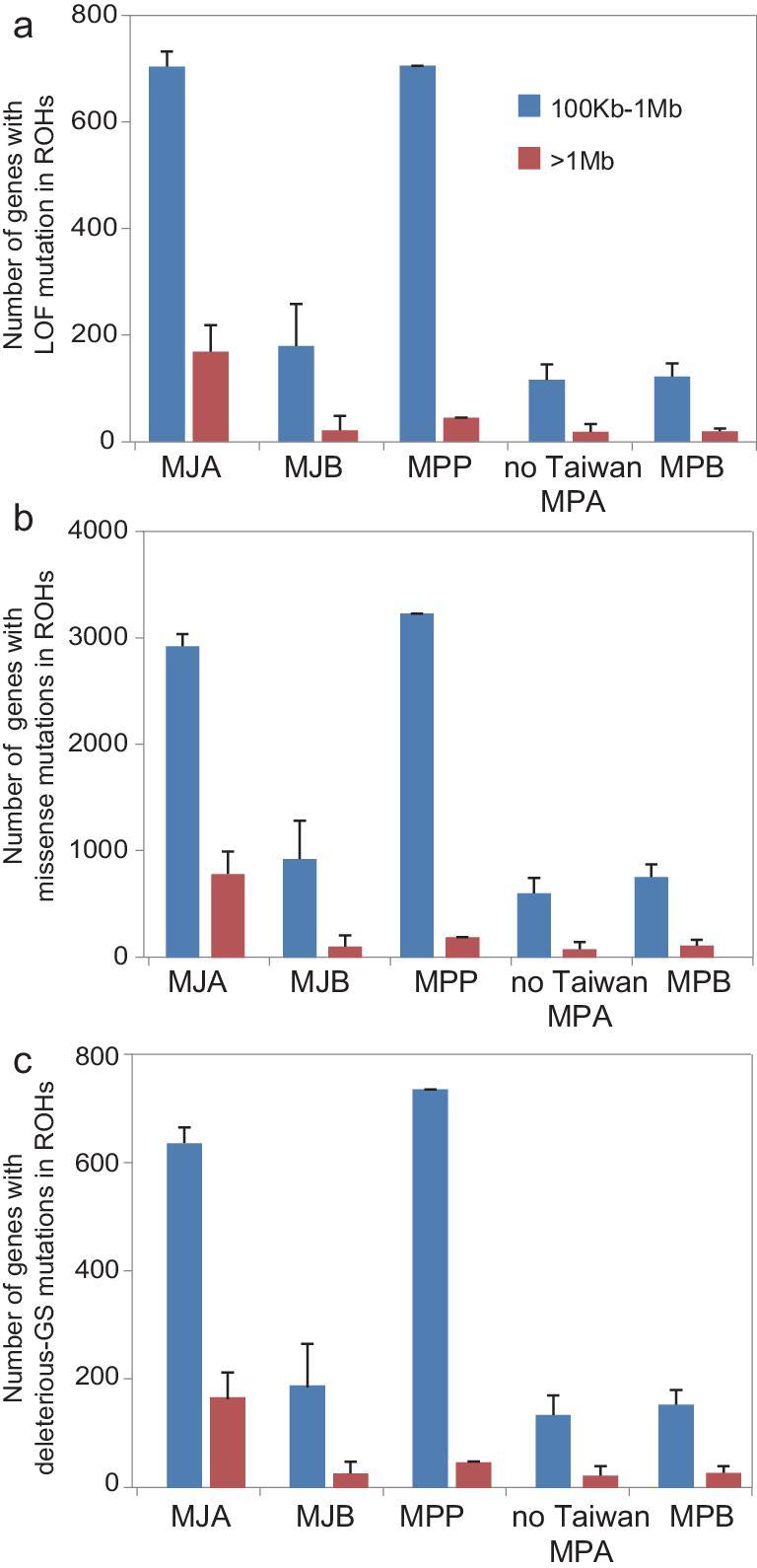
The number of genes with homozygous deleterious mutations in ROHs, including LOF mutations (a), missense mutations (b) and deleterious-GS mutations (c), of MJA, MJB, MPP, non-Taiwan MPA and MPB. Error bars represent SD.

Next, we assessed the genetic load in the MJA and MPP populations by identifying putative deleterious loss-of-function (LOF) and missense variants (Fig. 6c). The mutation of homozygous-derived alleles under evolutionary constraints is measured as an indicator of genetic load and predicted as approximate fitness consequences [37,52–54]. The results showed that the ratio of the number of homozygous sites to the homozygous and heterozygous sites in the MJA population was significantly higher than in the MJB population (0.948 vs. 0.908 for LOF; 0.855 vs. 0.800 for missense mutations; Wilcox-test, *P* < 0.001) (Fig. 6c). A similar difference was observed when comparing the Taiwan pangolin with other MPA individuals (0.947 vs. 0.917 for LOF; 0.883 vs. 0.838 for missense mutations; *P *< 0.001) and the MPB population (0.947 vs. 0.917 for LOF; 0.883 vs. 0.827 for missense mutations; *P *< 0.001) (Fig. 6c). In addition, the derived deleterious missense variants were predicted by Grantham Score (≥150) [55] that measured the physical and chemical properties of amino-acid changes. We also found an excess of homozygous deleterious-derived mutations in MJA and MPP (Fig. 6c). Therefore, the genetic load varied among populations, with a significantly higher genetic load in the MJA and MPP populations than in other populations. Interestingly, we found that the number of genes with homozygous deleterious mutations in ROHs was significantly higher in the MJA population than those in the MJB population (*P *< 0.001; Fig. 7). A similar difference was observed when comparing the Taiwan pangolin with other MPA individuals and the MPB population (*P *< 0.01; Fig. 7). Therefore, the increased genetic load was likely due to the inbreeding. Although MPP was the only Chinese pangolin population that has increased over the past two decades as a result of successful conservation efforts [56], our result indicated that the genetic diversity in this population was very low and the risk of inbreeding will need to be addressed in the future. We suggest that future conservation efforts could consider the possibility of rescuing this population by introducing mainland pangolins (e.g. from the MPA population) to increase the genetic diversity and adaptive potential of Taiwan pangolins.

We analyzed the genes affected by the homozygous putative deleterious LOF mutations [52] in MJA and MPP, and found that a number of Gene Ontology (GO) and Kyoto Encyclopedia of Genes and Genomes (KEGG) categories were involved in processes relating to cancer, disease and metabolism (Supplementary Table 11). For example, LOF mutations were found to enrich in the cholesterol homeostasis (Gene Ontology term, GO0042632) in MJA (Supplementary Table 11). The insufficient or excessive cholesterol has serious cellular consequences and leads to metabolic syndrome and cardiovascular diseases [57]. Pangolins live on a unique diet of high-cholesterol ants and termites [17,24,25]. The LOF mutations in genes related to cholesterol homeostasis may confer increased susceptibility to diseases due to abnormal cholesterol levels, which could be caused by a decrease in the utilization of food, leading to malnutrition and loss of competitiveness in the community.

## CONCLUSION

In this study, we recovered the population identities of illegally traded pangolins and revealed the previously unrecognized genetic lineages, including a Malayan pangolin population that might be continentally distributed (MJA in Fig. 2) and a Chinese pangolin population that might originate from Myanmar (MPB in Fig. 2) that should be protected and managed as evolutionarily distinct conservation units. The ancient demographic history analyses showed the effects of long-term environmental changes, including climate and sea-level changes. The ROHs and genetic load analyses demonstrated the increased inbreeding and genetic load effect of recent population decline due to recent human activities. Considering the short generation time and the small population size of pangolins, it would be interesting to investigate museum pangolin specimens, which could further quantify the impacts of human activities. In the future, it would be essential to establish population-specific genetic fingerprints of pangolins across the distribution of pangolins. The population-specific genetic fingerprints would be very useful to trace the origin of illegally traded pangolins, which will ultimately support long-term pangolin conservation efforts.

### A note added after acceptance

By coincidence, the acceptance of this study coincides with the COVID-19 outbreak in China. It has been widely reported that the causative coronavirus (SARS-CoV-2) may have been passed from the primary host (bats) to humans via pangolins as the secondary host. We have recently obtained the RNA-seq data from several tissues of a Malayan pangolin individual and made them available to the public (deposited under project in NCBI: PRJNA531293/NGDC: PRJCA002221). Our preliminary analysis of the five tissues (large intestine, small intestine, stomach, lung and cerebrum) of this pangolin individual did not show any evidence of the SARS-CoV-2 virus. While a comprehensive survey will be carried out by adding more tissues from more pangolin individuals (as well as other wild animals), this preliminary analysis suggests that SARS-CoV-2 viruses should not be expected to exist in all pangolins, if they indeed do.

## METHODS

### Sample collection

All necessary research permits and ethics approvals were granted in this study. In total, 73 Malayan pangolin samples between 2000 and 2017 and 22 Chinese pangolin samples between 1990 and 2017 were collected by the Animal Branch of the Germplasm Bank of Wild Species of Chinese Academy of Sciences, Guangzhou Wildlife Rescue Center and Nujiang Forest Public Security Bureau. Eleven pangolins were sampled from Yunnan Province, China and one from Kachin, Myanmar. Others were from seizures of illegal wildlife trade with unknown sampling locations. Detailed sample information is provided in Supplementary Table 3.

In addition, the short-read library reads from a Malayan pangolin individual (from Malaysia) and a Chinese pangolin individual (from Taiwan) [16] were included in our analyses.

### Species identification

Genomic DNA was extracted from the collected specimens using a QIAamp DNA Tissure Mini Kit (Qiagen, USA). Pangolin species identification of the specimens was confirmed by using a 658-bp region of mt cytochrome c oxidase subunit I (*COI*) gene with two pairs of mixed primer sets LepF1/LepR1 and VF1d/VR1d [58]. Polymerase chain reaction (PCR) amplification conditions were initial denaturation (98°C) for 2 min, followed by 35 cycles of denaturation (10 s at 98°C), annealing (15 s at 58°C) and extension (1 min at 72°C) and a final extension at 72°C (5 min). The PCR products were sequenced using LepF1 and LepR1. The generated *COI* sequences were checked carefully and queried in BLAST searches of GenBank to identify the pangolin species. *COI* sequences for species identification have been deposited in GenBank with accession numbers MK810451–MK810545.

### *De novo* genome sequencing and assembly

DNA samples from a Malayan pangolin (MJ74) and a Chinese pangolin (MP20) that was confiscated in Yunnan Province were used for *de novo* sequencing (Supplementary Tables 1–3). A whole-genome shotgun sequencing strategy was applied. Eleven libraries consisting of 4 short paired-end inserts (250 and 500 bp) and 8 long mate-paired inserts (2k∼15k) for a Chinese pangolin and 36 libraries consisting of 4 short paired-end inserts (220 and 500 bp) and 32 mate-paired inserts (3k∼18k) for a Malayan pangolin were constructed (Supplementary Table 1). In addition, 10X Genomes libraries for both pangolins were constructed (Supplementary Table 1). All libraries were sequenced on the Illumina HiSeq platform.

All the Illumina paired-end reads were trimmed for adaptor sequence and the reads containing >10% ambiguous nucleotides and >20% low-quality nucleotides (Q ≤ 5) were discarded. The average sequence error rate for each base was 0.04% (see Supplementary Table 1). The filtered Illumina paired-end reads were assembled into scaffolds using ALLpaths-LG [59] and the gaps were then filled using GapCloser v.1.12 [60]. Finally, the 10X linked-reads were used to link the scaffolds using fragScaff [61].

To assess the quality of the genome assembly, the short insert library reads were mapped onto the assembled genome using a Burrows-Wheeler Aligner (BWA) v.0.7.12 [62] to calculate the mapping ratio and assess the assembly integrity. In addition, CEGMA [27] and BUSCO [28] pipelines were used to validate the completeness of the assembly. Two genome assemblies have been deposited in NCBI under projects PRJNA529512 and PRJNA529513.

### Repeat identification, gene prediction and annotation

Repeats of the Malayan- and Chinese-pangolin genomes were identified using RepeatMsker [63], the Repbase library [63] and RepeatProteinMask [63]. The *de novo* repeat annotation was performed using LTR_FINDER [64], RepeatScout [65], RepeatModeler [66] and TRF [67].

Using the repeat-masked genome, we predicted genes by combining three approaches. First, AUGUSTUS [68], Glimmer-HMM [69], SNAP [70], Geneid [71] and Genscan [72] were used for *ab initio* prediction. Second, we assembled the RNA-seq reads from the tissues of a Malayan pangolin individual into transcripts using Tophat [73] and Cufflinks [74] for gene-structure annotation. Thirteen tissues (large intestine, small intestine, stomach, liver, pancreas, lung, ovary, cerebrum, tongue, kidney, heart, eyes and muscle) of the Malayan pangolin, which unfortunately died in the process of rescue, were obtained from Guangzhou Wildlife Rescue Center. Total RNA was extracted from each tissue using the TRIzol kit (Life Technologies). Libraries were constructed and sequenced according to the Illumina protocol and the average size of sequencing raw data was 6.98 Gb (deposited under project in NCBI: PRJNA531293/NGDC: PRJCA002221). Third, we used BLAST (E-value ≤$1.0{\rm{\ }} \times {10^{ - 5}}$) [75] and Genewise [76] to align the protein sequences from *Homo sapiens*, *Mus musculus*, *Canis familiaris*, *Felis catus*, *Capra hircus*, *Equus caballus* and *Myotis brandtii* to the Malayan pangolin and Chinese pangolin genomes for homology-based gene prediction. The gene sets predicted by the three approaches were integrated using EvidenceModeler [77] to produce consensus gene models, which were then updated by PASA [78].

### Genome re-sequencing of Malayan and Chinese pangolin populations

Libraries with an insert size of 500 bp were constructed to re-sequence Malayan and Chinese pangolin individuals according to the Illumina protocol. All libraries were sequenced on the Illumina HiSeq platform. All the Illumina paired-end reads were trimmed for adaptor sequence and the reads containing >10% ambiguous nucleotides and >20% low-quality nucleotides (Q ≤ 5) were discarded; 95.79% of the nucleotide sites in the filtered reads were of high quality, with a phred score $ \ge 20$, and 91.73% of the sites had a phred score $ \ge 30$. The average sequence error rate for each base was 0.03% (see Supplementary Table 3).

In addition, the short-read library reads were also collected from a previously published *de novo* Malayan pangolin individual (from Malaysia; NCBI, SRA, SRR3949728) and a Chinese pangolin individual (from Taiwan; NCBI, SRA, SRR770330-SRR770587) [16] as well as those from the two newly sequenced *de novo* Malayan and Chinese pangolin individuals in this study. In total, 74 Malayan and 23 Chinese pangolins were used for population genomic analyses (Supplementary Table 3). Re-sequencing data are available at NCBI Read Archive under project PRJNA529540.

### Re-sequencing data processing and SNP calling

We used BWA v.0.7.12 [62] to align the trimmed paired-end reads from the Malayan pangolin individual and the Chinese pangolin individual to our *de novo* assembled Malayan and Chinese pangolin reference genomes, respectively. Binary sequence alignment files were generated using SAMtools v.1.3 [79]. PCR duplicates were removed using PICARD (http://picard.sourceforge.net). Insert/InDel (insert and deletions) realignment was performed using the IndelRealigner algorithm implemented in the Genome Analysis Toolkit (GATK) v.3.5.0 [80].

Before conducting the SNP-calling analyses, the candidate sex-chromosome scaffolds from the assembled genomes that show >60% sequence similarity with sex chromosomes of *Canis lupus familiaris* (Chr X from CanFam3.1 [81]; Chr Y from KP081776.1 [82]) and those of *Homo sapiens* (GRCh38.p12) [83] were excluded by LASTZ (http://www.bx.psu.edu/∼rsharris/lastz/). The autosomal scaffolds were used in the subsequent analyses. In addition, the scaffolds with length <100 kb were excluded.

We identified autosomal SNPs using SAMtools mpileup [79]. SNPs with an allele frequency <20% of each individual and with a depth distribution of all sites <2.5% or >97.5% were filtered using a custom script to obtain high-quality SNPs. Moreover, low-quality SNPs were filtered out when the base-quality and mapping-quality score was <20.

### Haplotype network analyses based on mt DNA

More than 1 million pangolins have been poached in recent years. It is important to trace the original locations of illegally traded pangolins. The most available molecular data of pangolins are from mt DNA. Previous studies have reported concatenated mt *COI* and *CYTB* analyses (1575 bp) of Malayan pangolins with known and assigned geographic sources from Southeast Asia [12,15,23], so we extracted the *COI* and *CYTB* sequences from our genomic sequences. The *COI* and *CYTB* sequences of Malayan pangolins in this study were determined using BWA [62] by mapping the short reads to the reference mt sequences of Malayan pangolins (GenBank accession number KT445979). The consensus *COI* and *CYTB* sequences between the mapped short reads and the reference mt sequence were extracted using SAMtools [79] and an in-house Perl script. The concatenated *COI* and *CYTB* sequences of 74 Malayan pangolins in this study and those of another 60 individuals in previous studies [12,15,23] were generated.

The same strategy was also applied to Chinese pangolins. We downloaded the sequences of the *COI* gene (600 bp) from 10 individuals sampled from Guangdong, Hunan, Hainan and Taiwan in China and Thailand [6,9,12] and the *CYTB* gene (782 bp) from 15 individuals sampled from Taiwan in China and Thailand [6,12]. The reference mt sequence (GenBank accession number MG196307) was used. In total, the *COI* data set from 33 Chinese pangolins and the *CYTB* data set from 38 Chinese pangolins were obtained.

The resulting data sets were aligned using ClustalW [84] and the ambiguous sites were removed using Gblocks (gap = all) [85]. We generated a haplotype network using the Median-Joining method in PopART [86].

### Phylogenetic tree reconstruction based on nuclear genomes

To build a genome tree based on autosomal SNPs data sets, we first converted VCF files to FASTA files and then constructed an NJ tree using MEGA X [87] with the Kimura's two-parameter model. To validate the converting process, we also built the genome NJ tree from the VCF file using the eGPS software [88] with the same substitution model. We also constructed an ML tree using SNPhylo [89]. One thousand bootstraps were performed for both trees. SNPhylo is a newly developed pipeline for constructing phylogenetic trees based on large SNP data sets and has steps for removing low-quality data and considering LD to increase the reliability of a tree. We first used SNPhylo with parameter -p 25 to generate sequences from the extracted representative SNPs and then reconstructed an ML tree by running the RAxML v.8.2.4 program [90] with the GTRGAMMA model.

### Population structure and admixture analyses

The autosomal SNPs were thinned by LD values using PLINK v.1.1 [91], resulting in a set of ∼1.13- and ∼0.42-Mb SNPs for the population-structure and admixture analyses of MJ and MP, respectively. PCA was carried out using the smartPCA program from the Eigensoft package [92] and the Tracy-Widom test. Admixture analysis was performed using Frappe v.1.1 [93]. The postulated number of ancestral clusters (K) was set from 2 to 5 and the maximum number of expectation-maximization iterations was set to 10 000. The best K value was identified using the cross-validation procedure by ADMIXTURE [94].

### Demographic history analyses

We used the maximum-composite-likelihood approach based on the joint SFS implemented in fastsimcoal2 [95] to assess the fit of various demographic models and to infer the final optimal demographic scenario of the Malayan and Chinese pangolins. The scripts for the demographic modeling are available upon the request.

First, the joint SFS of different populations was inferred by assuming the allele state of the out-group as the ancestral allele state and removing the heterozygote allele sites in the out-group genome. The two newly sequenced *de novo* Malayan and Chinese pangolin genomes were used as the out-group genomes for each other's data sets. A total of 27 735 083 SNPs and 36 739 658 SNPs were used for the Malayan pangolin data set and Chinese pangolin data set analyses, respectively. The mutation rate per generation was set as 1.47 × 10^−8^ [16] and the generation time as 1 year [96,97].

In each parameter-estimation procedure, 100 000 coalescent simulations and ≥20 expectation-conditional maximization cycles, up to a maximum of 40, were used. To get a reliable global maximum estimation for each scenario and avoid a local maximum, we ran between 200 and 300 replicates. The AIC [98] was used to compare the fit of different models. The preferred point estimations of all parameters, including the migration rate, divergence time and effective population size, were chosen by the ML method and their confidence interval (CI) was calculated by simulating 100 independent site-frequency spectra conditional on the preferred demographic scenario. Re-estimation procedures were implemented for each simulated data set at least twice. We further validated our results using the SFS analyses (Supplementary Fig. 2) and the genetic-diversity comparison between the simulation and observed data set (Supplementary Fig. 3 and Supplementary Table 10).

In addition, we also reconstructed the demographic history of the Taiwan pangolin, which is of specific interest in this study as an island-endemic pangolin compared to their continental relatives among the Chinese pangolins. We used the multiple-sequential Markovian coalescent model [99] based on the phased haplotypes to estimate the divergence time and demographic history of the Taiwan pangolin. The haplotypes were phased using BEAGLE v.5.0 [100]. The mutation rate (μ) and the generation time were set as the same as those described above.

### Genetic diversity, ROH and LD analyses

The heterozygosity, which was used to assess the genetic diversity, was calculated based on autosomal SNPs with a 50-kb non-overlapping sliding-window size using VCFtools [101]. To test whether the resulting higher heterozygosity was caused by the higher sequence coverage, we subsampled the sequence reads from the MP *de novo* genome to the mean depth in MJ (15 X) and found a higher heterozygosity of MP (0.128%) than that of MJ (0.085%). Therefore, the higher heterozygosity of MP was not caused by the higher sequence coverage of MP.

PLINK [91] was used to identify the ROH in each pangolin following the method described before [31]. Only filtered ROHs that are >10 kb and contain <20 SNPs were taken into consideration. The extreme 5% ROH differences between MJ and MP were longer than 2.5 Mb (Supplementary Fig. 4). Using the physical length of the ROH as approximation for the genetic length [52], the ROH of a 2.5-Mb trace (g = 100/2 ^*^ ROH_length_, where g is the number of generations and ROH_length_ is the ROH length in centimorgans) was estimated to occur <20 generations ago.

The LD was calculated using Haploview [102]. We calculated the r square statistic (*r*^2^), which is the correlation coefficient between two focal loci of interest.

### Deleterious mutation patterns

Deleterious mutations are expected to disrupt gene function or reduce an individual's viability [32]. We used SnpEff v.4.3t [103] to categorize the derived allele mutations of the coding regions of each individual into missense and LOF mutations. Similarly to Feng *et al.*’s strategy [53], the genotypes of major homozygous alleles in each pangolin species (>50% of the individuals) and also the same homozygous alleles in the other pangolin species (as the out-group) were used to represent the ancestral state. We built the databases from the annotations and the reference-genome sequences of the two pangolin species. The input files in VCF format were used to annotate SNPs and we assigned a mutation category to the input SNPs per individual. LOF mutations included premature stop codons (nonsense) and splice-site-disrupting single-nucleotide variants. The ratio of the number of homozygous sites (two per site) to both homozygous and heterozygous sites (two per homozygous site and one per heterozygous site) for each category was evaluated for estimating the deleterious load [32,53].

The deleteriousness of the missense mutations was also diagnosed using the Grantham Score (GS) [55]—a measure of the physical/chemical properties of amino-acid changes. Grantham scores ≥150 were designated as deleterious [104].

For all identified homozygous LOF mutations (with an absolute allele frequency difference between populations >0.2) [52], we obtained the associated genes based on the human-genome annotation using BLASTP (E-value $ \le 1.0{\rm{\ }} \times {10^{ - 10}}$) [75]. The GO and KEGG functional enrichment of genes affected by those LOF mutations was assessed based on the DAVID database [105]. To avoid the influence of assembly and annotation artifacts on the LOF results, we also confirmed these results by comparing them with the two genomes independently published previously [16].

## Supplementary Material

nwaa031_Supplemental_File
