# Supplementary material for: Genomic consequences of population decline in critically endangered pangolins and their demographic histories
Source: Natl Sci Rev. 2020 Feb 27;7(4):798–814. doi: 10.1093/nsr/nwaa031 (PMC8288997; doi:10.1093/nsr/nwaa031)
Supplement: nwaa031_Supplemental_File [file nwaa031_supplemental_file.docx]

**Table S1 The information of NGS library and sequencing data set for MJ and MP.**

**Table S2 Sequencing data summary of two updated pangolin reference genomes and the comparisons with previously published pangolin genomes.**

**Table S3 Sample information and sequencing data summary of 74 Malayan pangolin (MJ) individuals and 23 Chinese pangolin (MP) individuals used in population genomic analyses.**

| **Sample ID** | **Seizure location^a^** | **Sample provider** | **Sampling time** | | **Clean data (bp)** | **Coverage (X)** | Error Rate  (%) | Q20(%) | Q30(%) | **Mapping rate (%)** | **Number of heterozygous SNPs** | **Number of homozygous SNPs** | **Heterozygosity** |
| --- | --- | --- | --- | --- | --- | --- | --- | --- | --- | --- | --- | --- | --- |
| MJ01 | Sino-Burmese border | Animal Branch of the Germplasm Bank of Wild Species, Chinese Academy of Sciences, China | 2014 | | 35,433,748,250 | 14.89 | 0.05 | 92.13 | 85.5 | 96.93 | 795,259 | 19,222,187 | 3.95E-04 |
| MJ02 | Sino-Burmese border | Animal Branch of the Germplasm Bank of Wild Species, Chinese Academy of Sciences, China | 2014 | | 35,259,629,500 | 14.81 | 0.05 | 93.16 | 87.75 | 95.97 | 861,461 | 19,155,985 | 4.15E-04 |
| MJ03 | Sino-Burmese border | Animal Branch of the Germplasm Bank of Wild Species, Chinese Academy of Sciences, China | 2014 | | 31,164,428,750 | 13.09 | 0.04 | 94.78 | 90.19 | 95.83 | 867,027 | 19,150,419 | 4.34E-04 |
| MJ04 | Sino-Burmese border | Animal Branch of the Germplasm Bank of Wild Species, Chinese Academy of Sciences, China | 2014 | | 33,069,399,000 | 13.89 | 0.04 | 94.85 | 90.21 | 96.90 | 910,166 | 19,107,280 | 4.64E-04 |
| MJ05 | Sino-Burmese border | Animal Branch of the Germplasm Bank of Wild Species, Chinese Academy of Sciences, China | 2014 | | 32,926,722,250 | 13.83 | 0.04 | 94.1 | 88.94 | 96.49 | 753,956 | 19,263,490 | 4.63E-04 |
| MJ06 | Yunnan, China | Animal Branch of the Germplasm Bank of Wild Species, Chinese Academy of Sciences, China | 2016 | | 30,363,351,600 | 12.76 | 0.03 | 96.48 | 91.7 | 96.96 | 888,464 | 19,128,982 | 4.63E-04 |
| MJ07 | Yunnan, China | Animal Branch of the Germplasm Bank of Wild Species, Chinese Academy of Sciences, China | 2017 | | 44,586,773,100 | 18.73 | 0.02 | 97.44 | 92.82 | 96.83 | 843,752 | 19,173,694 | 4.01E-04 |
| MJ08 | Yunnan, China | Animal Branch of the Germplasm Bank of Wild Species, Chinese Academy of Sciences, China | 2017 | | 38,151,996,000 | 16.03 | 0.02 | 96.31 | 94.53 | 96.84 | 809,767 | 19,207,679 | 3.93E-04 |
| MJ09 | Yunnan, China^*^ | Animal Branch of the Germplasm Bank of Wild Species, Chinese Academy of Sciences, China | 2000 | | 35,935,236,750 | 15.10 | 0.04 | 95.01 | 90.74 | 96.99 | 860,890 | 19,156,556 | 4.11E-04 |
| MJ10 | Sino-Burmese border | Animal Branch of the Germplasm Bank of Wild Species, Chinese Academy of Sciences, China | 2014 | | 37,658,480,750 | 15.82 | 0.04 | 95.62 | 91.67 | 96.87 | 884,314 | 19,133,132 | 4.18E-04 |
| MJ11 | Yunnan, China | Animal Branch of the Germplasm Bank of Wild Species, Chinese Academy of Sciences, China | 2017 | | 34,530,873,000 | 14.51 | 0.02 | 96.16 | 94.32 | 97.02 | 891,017 | 19,126,429 | 4.45E-04 |
| MJ12 | Sino-Burmese border | Animal Branch of the Germplasm Bank of Wild Species, Chinese Academy of Sciences, China | 2014 | | 36,419,284,750 | 15.30 | 0.04 | 95.51 | 91.51 | 96.99 | 921,202 | 19,096,244 | 4.41E-04 |
| MJ13 | Yunnan, China | Animal Branch of the Germplasm Bank of Wild Species, Chinese Academy of Sciences, China | 2017 | | 32,467,806,600 | 13.64 | 0.02 | 97.44 | 92.85 | 96.99 | 788,525 | 19,228,921 | 4.12E-04 |
| MJ14 | Sino-Burmese border | Animal Branch of the Germplasm Bank of Wild Species, Chinese Academy of Sciences, China | 2014 | | 42,888,551,000 | 18.02 | 0.04 | 94.2 | 89.24 | 97.09 | 846,374 | 19,171,072 | 3.95E-04 |
| MJ15 | Sino-Burmese border | Animal Branch of the Germplasm Bank of Wild Species, Chinese Academy of Sciences, China | 2014 | | 37,421,435,500 | 15.72 | 0.06 | 92.43 | 86.56 | 97.42 | 895,564 | 19,121,882 | 6.16E-04 |
| MJ16 | Yunnan,China^*^ | Animal Branch of the Germplasm Bank of Wild Species, Chinese Academy of Sciences, China | 2000 | | 35,522,948,500 | 14.93 | 0.04 | 94.74 | 90.05 | 96.99 | 871,659 | 19,145,787 | 4.31E-04 |
| MJ17 | Yunnan,China^*^ | Animal Branch of the Germplasm Bank of Wild Species, Chinese Academy of Sciences, China | 2000 | | 36,983,489,750 | 15.54 | 0.04 | 95.19 | 90.81 | 97.01 | 862,768 | 19,154,678 | 4.08E-04 |
| MJ18 | Sino-Burmese border | Animal Branch of the Germplasm Bank of Wild Species, Chinese Academy of Sciences, China | 2014 | | 33,543,833,750 | 14.09 | 0.04 | 94.73 | 90.05 | 96.80 | 824,733 | 19,192,713 | 4.10E-04 |
| MJ19 | Sino-Burmese border | Animal Branch of the Germplasm Bank of Wild Species, Chinese Academy of Sciences, China | 2014 | | 35,769,621,250 | 15.03 | 0.05 | 92.65 | 86.44 | 96.94 | 886,666 | 19,130,780 | 4.33E-04 |
| MJ20 | Yunnan, China | Animal Branch of the Germplasm Bank of Wild Species, Chinese Academy of Sciences, China | 2017 | | 37,886,220,600 | 15.92 | 0.02 | 95.92 | 93.445 | 95.62 | 2,454,275 | 17,563,171 | 1.26E-03 |
| MJ21 | Sino-Burmese border | Animal Branch of the Germplasm Bank of Wild Species, Chinese Academy of Sciences, China | 2014 | | 30,352,321,250 | 12.75 | 0.05 | 93.09 | 87.19 | 88.71 | 878,957 | 19,138,489 | 4.84E-04 |
| MJ22 | Sino-Burmese border | Animal Branch of the Germplasm Bank of Wild Species, Chinese Academy of Sciences, China | 2014 | | 38,855,989,000 | 16.33 | 0.03 | 95.59 | 91.62 | 96.98 | 801,201 | 19,216,245 | 3.77E-04 |
| MJ23 | Sino-Burmese border | Animal Branch of the Germplasm Bank of Wild Species, Chinese Academy of Sciences, China | 2014 | | 37,223,682,000 | 15.64 | 0.04 | 94.79 | 90.13 | 96.68 | 845,317 | 19,172,129 | 4.02E-04 |
| MJ24 | Sino-Burmese border | Animal Branch of the Germplasm Bank of Wild Species, Chinese Academy of Sciences, China | 2014 | | 35,810,776,250 | 15.00 | 0.04 | 93.51 | 88.2 | 97.04 | 817,456 | 19,199,990 | 4.80E-04 |
| MJ25 | Sino-Burmese border | Animal Branch of the Germplasm Bank of Wild Species, Chinese Academy of Sciences, China | 2014 | | 33,646,914,250 | 14.14 | 0.05 | 92.26 | 85.68 | 96.97 | 793,921 | 19,223,525 | 3.95E-04 |
| MJ26 | Myanmar**^△^** | Nujiang Forestry Bureau,Yunnan, China | 2017 | | 39,480,716,100 | 16.59 | 0.03 | 97.4 | 95.92 | 96.64 | 778,785 | 19,238,661 | 3.70E-04 |
| MJ27 | Yunnan, China | Animal Branch of the Germplasm Bank of Wild Species, Chinese Academy of Sciences, China | 2017 | | 43,215,808,500 | 18.16 | 0.02 | 97.45 | 92.91 | 97.28 | 700,298 | 19,317,148 | 3.36E-04 |
| MJ28 | Sino-Burmese border | Animal Branch of the Germplasm Bank of Wild Species, Chinese Academy of Sciences, China | 2014 | | 34,373,986,500 | 14.44 | 0.04 | 94.79 | 90.11 | 96.73 | 807,231 | 19,210,215 | 4.02E-04 |
| MJ29 | Yunnan, China | Animal Branch of the Germplasm Bank of Wild Species, Chinese Academy of Sciences, China | 2017 | | 35,915,603,700 | 15.09 | 0.02 | 96.24 | 93.865 | 98.11 | 747,133 | 19,270,313 | 3.63E-04 |
| MJ30 | Sino-Burmese border | Animal Branch of the Germplasm Bank of Wild Species, Chinese Academy of Sciences, China | 2014 | | 34,157,131,250 | 14.35 | 0.05 | 92.75 | 86.55 | 96.98 | 888,720 | 19,128,726 | 4.59E-04 |
| MJ31 | Yunnan, China | Animal Branch of the Germplasm Bank of Wild Species, Chinese Academy of Sciences, China | 2017 | | 31,245,751,200 | 13.13 | 0.02 | 97.36 | 92.77 | 97.96 | 869,754 | 19,147,692 | 5.30E-04 |
| MJ32 | Sino-Burmese border | Animal Branch of the Germplasm Bank of Wild Species, Chinese Academy of Sciences, China | 2014 | | 39,561,439,750 | 16.62 | 0.04 | 93.39 | 88.01 | 97.00 | 829,551 | 19,187,895 | 4.41E-04 |
| MJ33 | Sino-Burmese border | Animal Branch of the Germplasm Bank of Wild Species, Chinese Academy of Sciences, China | 2014 | | 30,442,583,250 | 12.79 | 0.05 | 92.51 | 86.22 | 95.80 | 873,924 | 19,143,522 | 4.84E-04 |
| MJ34 | Sino-Burmese border | Animal Branch of the Germplasm Bank of Wild Species, Chinese Academy of Sciences, China | 2014 | | 30,735,027,000 | 12.91 | 0.04 | 94.635 | 89.98 | 96.76 | 853,160 | 19,164,286 | 4.53E-04 |
| MJ35 | Sino-Burmese border | Animal Branch of the Germplasm Bank of Wild Species, Chinese Academy of Sciences, China | 2014 | | 28,849,679,500 | 12.12 | 0.05 | 92.93 | 86.94 | 96.29 | 827,892 | 19,189,554 | 4.88E-04 |
| MJ36 | Sino-Burmese border | Animal Branch of the Germplasm Bank of Wild Species, Chinese Academy of Sciences, China | 2014 | | 31,034,308,500 | 13.04 | 0.05 | 92.57 | 86.2 | 96.85 | 869,750 | 19,147,696 | 4.45E-04 |
| MJ37 | Yunnan, China | Animal Branch of the Germplasm Bank of Wild Species, Chinese Academy of Sciences, China | 2017 | | 41,001,467,700 | 17.23 | 0.02 | 97.46 | 92.88 | 97.00 | 906,241 | 19,111,205 | 4.39E-04 |
| MJ38 | Yunnan, China | Animal Branch of the Germplasm Bank of Wild Species, Chinese Academy of Sciences, China | 2017 | | 39,551,565,600 | 16.62 | 0.02 | 95.88 | 92.84 | 96.88 | 841,038 | 19,176,408 | 4.03E-04 |
| MJ39 | Yunnan, China | Animal Branch of the Germplasm Bank of Wild Species, Chinese Academy of Sciences, China | 2017 | | 35,464,207,500 | 14.90 | 0.02 | 97.18 | 92.25 | 97.05 | 844,082 | 19,173,364 | 4.26E-04 |
| MJ40 | Yunnan, China | Animal Branch of the Germplasm Bank of Wild Species, Chinese Academy of Sciences, China | 2017 | | 47,719,927,200 | 20.05 | 0.02 | 96.02 | 93.05 | 96.76 | 875,726 | 19,141,720 | 4.11E-04 |
| MJ41 | Sino-Burmese border | Animal Branch of the Germplasm Bank of Wild Species, Chinese Academy of Sciences, China | 2014 | | 31,597,040,750 | 13.28 | 0.04 | 95.26 | 91.09 | 96.77 | 914,915 | 19,102,531 | 4.59E-04 |
| MJ42 | Sino-Burmese border | Animal Branch of the Germplasm Bank of Wild Species, Chinese Academy of Sciences, China | 2014 | | 35,457,197,500 | 14.90 | 0.04 | 94.95 | 90.47 | 93.86 | 713,856 | 19,303,590 | 3.60E-04 |
| MJ43 | Yunnan, China | Animal Branch of the Germplasm Bank of Wild Species, Chinese Academy of Sciences, China | 2017 | | 34,150,572,900 | 14.35 | 0.02 | 97.15 | 92.27 | 96.83 | 1,896,001 | 18,121,445 | 1.00E-03 |
| MJ44 | Yunnan, China | Animal Branch of the Germplasm Bank of Wild Species, Chinese Academy of Sciences, China | 2017 | | 37,077,989,400 | 15.58 | 0.02 | 96.13 | 93.44 | 95.69 | 2,415,402 | 17,602,044 | 1.20E-03 |
| MJ45 | Guangzhou, China | Guangzhou wildlife rescue center, Guangzhou, China | 2017 | | 41,103,945,000 | 17.27 | 0.02 | 96.68 | 94.8 | 97.97 | 2,428,360 | 17,589,086 | 1.14E-03 |
| MJ46 | Guangzhou, China | Guangzhou wildlife rescue center, Guangzhou, China | 2018 | | 42,972,787,500 | 18.06 | 0.02 | 96.17 | 94.09 | 96.80 | 2,876,776 | 17,140,670 | 1.34E-03 |
| MJ47 | Yunnan, China | Animal Branch of the Germplasm Bank of Wild Species, Chinese Academy of Sciences, China | 2016 | | 31,773,359,100 | 13.35 | 0.03 | 95.47 | 89.3 | 96.49 | 2,628,496 | 17,388,950 | 1.31E-03 |
| MJ48 | Yunnan, China | Animal Branch of the Germplasm Bank of Wild Species, Chinese Academy of Sciences, China | 2003 | | 31,544,109,750 | 13.25 | 0.04 | 94.37 | 89.47 | 96.74 | 2,371,439 | 17,646,007 | 1.22E-03 |
| MJ49 | Yunnan, China | Animal Branch of the Germplasm Bank of Wild Species, Chinese Academy of Sciences, China | 2017 | | 31,414,188,300 | 13.20 | 0.03 | 96.54 | 91.37 | 98.30 | 2,639,855 | 17,377,591 | 1.35E-03 |
| MJ50 | Yunnan, China | Animal Branch of the Germplasm Bank of Wild Species, Chinese Academy of Sciences, China | 2016 | | 30,745,500,000 | 12.92 | 0.03 | 95.33 | 90.82 | 96.44 | 2,508,265 | 17,509,181 | 1.36E-03 |
| MJ51 | Yunnan, China | Animal Branch of the Germplasm Bank of Wild Species, Chinese Academy of Sciences, China | 2016 | | 30,336,713,100 | 12.75 | 0.03 | 97.005 | 92.66 | 96.82 | 3,033,734 | 16,983,712 | 1.77E-03 |
| MJ52 | Yunnan, China | Animal Branch of the Germplasm Bank of Wild Species, Chinese Academy of Sciences, China | 2016 | | 31,530,683,400 | 13.25 | 0.03 | 96.72 | 92.06 | 96.76 | 3,365,976 | 16,651,470 | 1.78E-03 |
| MJ53 | Yunnan, China | Animal Branch of the Germplasm Bank of Wild Species, Chinese Academy of Sciences, China | 2014 | | 30,012,719,700 | 12.61 | 0.03 | 96.96 | 92.555 | 96.95 | 2,742,312 | 17,275,134 | 1.50E-03 |
| MJ54 | Yunnan, China | Animal Branch of the Germplasm Bank of Wild Species, Chinese Academy of Sciences, China | 2017 | | 33,564,907,800 | 14.10 | 0.02 | 95.73 | 92.67 | 96.64 | 2,751,002 | 17,266,444 | 1.39E-03 |
| MJ55 | Yunnan, China | Animal Branch of the Germplasm Bank of Wild Species, Chinese Academy of Sciences, China | 2016 | | 37,085,506,500 | 15.58 | 0.03 | 97.41 | 93.4 | 96.89 | 2,567,234 | 17,450,212 | 1.27E-03 |
| MJ56 | Yunnan, China | Animal Branch of the Germplasm Bank of Wild Species, Chinese Academy of Sciences, China | 2017 | | 32,207,231,400 | 13.53 | 0.02 | 95.8 | 93.91 | 96.75 | 2,521,904 | 17,495,542 | 1.39E-03 |
| MJ57 | Yunnan, China | Animal Branch of the Germplasm Bank of Wild Species, Chinese Academy of Sciences, China | 2017 | | 34,821,522,900 | 14.63 | 0.02 | 96.41 | 94.11 | 96.58 | 2,558,874 | 17,458,572 | 1.26E-03 |
| MJ58 | Yunnan, China | Animal Branch of the Germplasm Bank of Wild Species, Chinese Academy of Sciences, China | 2016 | | 33,687,557,700 | 14.15 | 0.03 | 96.89 | 92.16 | 98.26 | 2,376,590 | 17,640,856 | 1.29E-03 |
| MJ59 | Yunnan, China | Animal Branch of the Germplasm Bank of Wild Species, Chinese Academy of Sciences, China | 2016 | | 36,260,783,100 | 15.24 | 0.03 | 97.29 | 93.16 | 96.99 | 2,583,168 | 17,434,278 | 1.29E-03 |
| MJ60 | Yunnan, China | Animal Branch of the Germplasm Bank of Wild Species, Chinese Academy of Sciences, China | 2016 | | 50,369,271,900 | 21.16 | 0.03 | 96.88 | 92.16 | 98.42 | 2,175,596 | 17,841,850 | 1.04E-03 |
| MJ61 | Yunnan, China | Animal Branch of the Germplasm Bank of Wild Species, Chinese Academy of Sciences, China | 2017 | | 36,411,399,900 | 15.30 | 0.02 | 95.7 | 93.7 | 97.15 | 856,612 | 19,160,834 | 4.10E-04 |
| MJ62 | Yunnan, China | Animal Branch of the Germplasm Bank of Wild Species, Chinese Academy of Sciences, China | 2016 | | 34,240,267,800 | 14.39 | 0.03 | 97.2 | 92.94 | 97.00 | 2,511,520 | 17,505,926 | 1.29E-03 |
| MJ63 | Yunnan, China | Animal Branch of the Germplasm Bank of Wild Species, Chinese Academy of Sciences, China | 2016 | | 38,552,650,800 | 16.20 | 0.01 | 97.62 | 94.5 | 97.69 | 3,229,952 | 16,787,494 | 1.65E-03 |
| MJ64 | Yunnan, China | Animal Branch of the Germplasm Bank of Wild Species, Chinese Academy of Sciences, China | 2017 | | 35,405,740,200 | 14.88 | 0.02 | 97.54 | 93.1 | 96.81 | 3,405,920 | 16,611,526 | 1.71E-03 |
| MJ65 | Yunnan, China | Animal Branch of the Germplasm Bank of Wild Species, Chinese Academy of Sciences, China | 2017 | | 44,279,100,900 | 18.60 | 0.02 | 95.91 | 92.9 | 96.79 | 2,772,683 | 17,244,763 | 1.31E-03 |
| MJ66 | Yunnan, China | Animal Branch of the Germplasm Bank of Wild Species, Chinese Academy of Sciences, China | 2017 | | 34,823,754,600 | 14.63 | 0.02 | 96.35 | 93.5 | 96.97 | 3,379,024 | 16,638,422 | 1.64E-03 |
| MJ67 | Yunnan, China | Animal Branch of the Germplasm Bank of Wild Species, Chinese Academy of Sciences, China | 2017 | | 29,393,499,000 | 12.35 | 0.02 | 95.89 | 92.87 | 97.05 | 2,654,269 | 17,363,177 | 1.40E-03 |
| MJ68 | Malaysia**^+^** | NCBI(SRR3949728) (Choo et al. 2016) | - | | 35,368,636,100^b^ | 14.86 | ~ | ~ | ~ | 96.61 | 2,985,147 | 17,032,299 | 1.43E-03 |
| MJ69 | Yunnan, China | Animal Branch of the Germplasm Bank of Wild Species, Chinese Academy of Sciences, China | 2017 | | 38,777,361,600 | 16.29 | 0.02 | 97.52 | 93.03 | 96.67 | 2,942,007 | 17,075,439 | 1.45E-03 |
| MJ70 | Guangzhou, China | Guangzhou wildlife rescue center, Guangzhou, China | 2018 | | 44,493,229,800 | 18.69 | 0.02 | 96.41 | 94.42 | 96.82 | 3,259,802 | 16,757,644 | 1.53E-03 |
| MJ71 | Guangzhou, China | Guangzhou wildlife rescue center, Guangzhou, China | 2,018 | | 37,339,387,500 | 15.69 | 0.02 | 96.28 | 94.22 | 96.74 | 3,261,191 | 16,756,255 | 1.57E-03 |
| MJ72 | Yunnan, China | Animal Branch of the Germplasm Bank of Wild Species, Chinese Academy of Sciences, China | 2015 | | 33,016,677,000 | 13.87 | 0.01 | 97.63 | 94.54 | 96.74 | 3,068,718 | 16,948,728 | 1.70E-03 |
| MJ73 | Yunnan, China | Animal Branch of the Germplasm Bank of Wild Species, Chinese Academy of Sciences, China | 2017 | | 34,497,275,700 | 14.49 | 0.02 | 97.23 | 92.41 | 96.74 | 3,184,672 | 16,832,774 | 1.66E-03 |
| MJ74 | Yunnan, China | Animal Branch of the Germplasm Bank of Wild Species, Chinese Academy of Sciences, China | 2014 | | 44,969,688,145^b^ | 18.89 | 0.04 | 92.13 | 86.39 | 96.67 | 3,083,965 | 16,933,481 | 1.52E-03 |
| MP01 | Yunnan, China^*^ | Animal Branch of the Germplasm Bank of Wild Species, Chinese Academy of Sciences, China | 1990 | | 119,095,165,000 | 41.50 | 0.01 | 97.865 | 95.05 | 99.01 | 2,937,898 | 18,879,990 | 1.29E-03 |
| MP02 | Yunnan, China^*^ | Animal Branch of the Germplasm Bank of Wild Species, Chinese Academy of Sciences, China | | 1992 | 105,120,030,800 | 36.63 | 0.01 | 97.525 | 94.255 | 98.82 | 2,932,813 | 18,885,075 | 1.29E-03 |
| MP03 | Yunnan, China^*^ | Animal Branch of the Germplasm Bank of Wild Species, Chinese Academy of Sciences, China | | 2000 | 110,647,100,900 | 38.55 | 0.03 | 97.23 | 93.515 | 98.60 | 2,893,236 | 18,924,652 | 1.27E-03 |
| MP04 | Yunnan, China^*^ | Animal Branch of the Germplasm Bank of Wild Species, Chinese Academy of Sciences, China | | 1992 | 103,230,134,500 | 35.97 | 0.04 | 95.97 | 92.16 | 98.97 | 2,928,687 | 18,889,201 | 1.29E-03 |
| MP05 | Yunnan, China^*^ | Animal Branch of the Germplasm Bank of Wild Species, Chinese Academy of Sciences, China | | 2000 | 130,604,772,450 | 45.51 | 0.02 | 97.72 | 94.41 | 98.07 | 2,965,581 | 18,852,307 | 1.30E-03 |
| MP06 | Yunnan, China^*^ | Animal Branch of the Germplasm Bank of Wild Species, Chinese Academy of Sciences, China | | 1990 | 111,001,919,000 | 38.68 | 0.015 | 97.43 | 94.145 | 98.71 | 2,842,071 | 18,975,817 | 1.25E-03 |
| MP07 | Taiwan, China**^○^** | NCBI(SRR770330-SRR770587) (Choo et al. 2016) | | - | 125,692,918,100^b^ | 43.79 | ~ | ~ | ~ | 98.06 | 910,740 | 20,907,148 | 4.00E-04 |
| MP08 | Yunnan, China^*^ | Animal Branch of the Germplasm Bank of Wild Species, Chinese Academy of Sciences, China | | 2000 | 121,869,125,200 | 42.46 | 0.015 | 97.935 | 94.975 | 98.44 | 3,124,742 | 18,693,146 | 1.37E-03 |
| MP09 | Yunnan, China | Animal Branch of the Germplasm Bank of Wild Species, Chinese Academy of Sciences, China | | 2017 | 115,164,624,300 | 40.13 | 0.02 | 97.38 | 92.79 | 98.69 | 3,178,536 | 18,639,352 | 1.40E-03 |
| MP10 | Yunnan, China | Animal Branch of the Germplasm Bank of Wild Species, Chinese Academy of Sciences, China | | 2017 | 109,073,589,300 | 38.00 | 0.02 | 97.34 | 92.81 | 99.27 | 3,153,822 | 18,664,066 | 1.46E-03 |
| MP11 | Yunnan, China^*^ | Animal Branch of the Germplasm Bank of Wild Species, Chinese Academy of Sciences, China | | 2005 | 113,421,686,500 | 39.52 | 0.02 | 98 | 94.9 | 98.40 | 3,116,941 | 18,700,947 | 1.37E-03 |
| MP12 | Yunnan, China | Animal Branch of the Germplasm Bank of Wild Species, Chinese Academy of Sciences, China | | 2017 | 96,074,054,100 | 33.48 | 0.02 | 96.39 | 94.65 | 99.05 | 2,963,089 | 18,854,799 | 1.31E-03 |
| MP13 | Yunnan, China | Animal Branch of the Germplasm Bank of Wild Species, Chinese Academy of Sciences, China | | 2016 | 114,888,722,400 | 40.03 | 0.03 | 97.03 | 93.015 | 99.04 | 3,587,986 | 18,229,902 | 1.58E-03 |
| MP14 | Yunnan, China | Animal Branch of the Germplasm Bank of Wild Species, Chinese Academy of Sciences, China | | 2017 | 93,086,067,600 | 32.43 | 0.02 | 95.62 | 92.5 | 98.26 | 3,679,812 | 18,138,076 | 1.62E-03 |
| MP15 | Yunnan, China | Animal Branch of the Germplasm Bank of Wild Species, Chinese Academy of Sciences, China | 2016 | | 116,580,735,600 | 40.62 | 0.025 | 97.495 | 93.96 | 99.44 | 3,537,000 | 18,280,888 | 1.55E-03 |
| MP16 | Sino-Burmese border | Animal Branch of the Germplasm Bank of Wild Species, Chinese Academy of Sciences, China | 2014 | | 101,065,501,250 | 35.21 | 0.04 | 93.88 | 89.3475 | 97.98 | 3,024,281 | 18,793,607 | 1.44E-03 |
| MP17 | Sino-Burmese border | Animal Branch of the Germplasm Bank of Wild Species, Chinese Academy of Sciences, China | 2014 | | 102,237,214,000 | 35.62 | 0.04 | 94.25 | 89.8275 | 99.01 | 2,771,842 | 19,046,046 | 1.23E-03 |
| MP18 | Sino-Burmese border | Animal Branch of the Germplasm Bank of Wild Species, Chinese Academy of Sciences, China | 2014 | | 117,100,735,250 | 40.80 | 0.04 | 95.32 | 91.09 | 98.85 | 2,766,484 | 19,051,404 | 1.21E-03 |
| MP19 | Yunnan, China | Animal Branch of the Germplasm Bank of Wild Species, Chinese Academy of Sciences, China | 2017 | | 104,129,231,700 | 36.28 | 0.02 | 97.39 | 92.77 | 99.24 | 2,757,948 | 19,059,940 | 1.21E-03 |
| MP20 | Yunnan, China | Animal Branch of the Germplasm Bank of Wild Species, Chinese Academy of Sciences, China | 2017 | | 145,683,756,000^b^ | 50.75 | 0.04 | 96.095 | 94.16 | 99.12 | 2,616,549 | 19,201,339 | 1.14E-03 |
| MP21 | Yunnan, China | Animal Branch of the Germplasm Bank of Wild Species, Chinese Academy of Sciences, China | 2016 | | 103,156,529,250 | 35.94 | 0.04 | 94.48 | 90.47416667 | 99.18 | 2,485,229 | 19,332,659 | 1.09E-03 |
| MP22 | Yunnan, China | Animal Branch of the Germplasm Bank of Wild Species, Chinese Academy of Sciences, China | 2016 | | 128,348,477,700 | 44.72 | 0.03 | 97.195 | 93.38 | 98.74 | 2,588,978 | 19,228,910 | 1.13E-03 |
| MP23 | Yunnan, China | Animal Branch of the Germplasm Bank of Wild Species, Chinese Academy of Sciences, China | 2017 | | 91,612,132,500 | 31.92 | 0.02 | 95.67 | 93.195 | 99.37 | 2,451,377 | 19,366,511 | 1.07E-03 |
| a: *○△+ represent the samples of known geographic location. | | | | | | | | | | | | | |
| b: The short-read library reads from a previously published *de novo* Malayan pangolin individual (from Malaysia; MJ68) and a Chinese pangolin individual (from Taiwan; MP07; Choo et al. 2016) as well as those from the two newly sequenced *de novo* Malayan (MJ74) and Chinese pangolin (MP20) individuals in this study were collected. | | | | | | | | | | | | | |

**Table S4 The genome-wide heterozygosity in 94 published mammals.**

| Species | Heterozygosity (%) | Sources |
| --- | --- | --- |
| Iberian lynx (*Lynx pardinus*) | 0.010 | Abascal et al. 2016 |
| Domestic cat (*Felis catus*) | 0.012 | Cho et al. 2013 |
| Baiji (*Lipotes vexillifer*) | 0.012 | Zhou et al. 2013 |
| myanmar snub-nosed monkey (*Rhinopithecus strykeri*) | 0.015 | Zhou et al. 2016 |
| Altai Neanderthal (*Homo sapiens*) | 0.017 | Prufer et al. 2014. |
| Cheetah (*Acinonyx jubatus*) | 0.020 | Dobrynin et al. 2015 |
| Snow leopard (*Panthera uncia syn*) | 0.023 | Cho et al. 2013 |
| Yangtze river dolphin (*Lipotes vexillifer*) | 0.026 | Zhou et al. 2013 |
| Eurasian lynx (*Lynx lynx*) | 0.028 | Abascal et al. 2016 |
| Siberian tiger (*Panthera tigris altaica)* | 0.030 | Dobrynin et al. 2015 |
| Domestic dog (*Canis familiaris*) | 0.032 | Lindblad-Toh et al. 2005 |
| Tasmanian devil (*Sarcophilus harrisii*) | 0.032 | Cho et al. Nat. 2013 |
| Black snub-nosed monkey (*Rhinopithecus bieti*) | 0.033 | Zhou et al. 2016 |
| San Miguel Island fox (*Urocyon littoralis*) | 0.033 | Robinson et al. 2016 |
| Bengal tiger (*Panthera tigris tigris*) | 0.040 | Dobrynin et al. 2015 |
| Golden snub-nosed monkey (*Rhinopithecus roxellana*) | 0.042 | Zhou et al. 2016 |
| White lion (*Panthera leo*) | 0.048 | Cho et al. 2013 |
| Amur tiger (*Panthera tigris altaica*) | 0.049 | Cho et al. 2013 |
| San Nicolis Island fox (*Urocyon littoralis*) | 0.049 | Robinson et al. 2016 |
| Aye-Aye (*Daubentonia madagascariensis*) | 0.051 | Perry et al. 2011 |
| Wild horse (*Equus ferus przewalskii*) | 0.052 | Huang et al. 2014 |
| Domestic Turkey (*Meleagris gallopavo*) | 0.057 | Dalloul et al. 2010 |
| African lion (*Panthera leo*) | 0.058 | Cho et al. 2013 |
| Cattle (*Bos taurus*) | 0.059 | Qiu et al. 2012 |
| grey snub-nosed monkey (*Rhinopithecus brelichi*) | 0.062 | Zhou et al. 2016 |
| Eastern lowland gorilla (*Gorilla beringei graueri*) | 0.064 | Xue et al. 2015 |
| Bornean orang-utan (*Pongo pygmaeus*) | 0.065 | Locke et al. 2011 |
| Mountain gorilla (*Gorilla beringei beringei*) | 0.065 | Xue et al. 2015 |
| Naked mole rat (*Heterocephalus glaber*) | 0.068 | Kim et al. 2011 |
| White tiger (*Panthera tigris tigris*) | 0.073 | Cho et al. 2013 |
| Pileated gibbon (*Hylobates pileatus*) | 0.073 | Carbone et al. 2014 |
| Dromedary (*Camelus dromedarius*) | 0.074 | Wu et al. 2014 |
| Platypus (*Ornithorhynchus anatinus*) | 0.075 | Warren et al. 2008 |
| Eastern lowland gorilla (*Gorilla beringei graueri*) | 0.076 | Scally et al. 2012 |
| Human_Han (*Homo species*) | 0.077 | Meyer et al. 2012 |
| West African chimpanzees (*Pan troglodytes verus*) | 0.080 | Mikkelsen et al. 2005 |
| African green monkey (*Chlorocebus aethiops aethiops*) | 0.080 | Warren et al. 2015 |
| Wild bactrian camel (*Camelus bactrianus ferus*) | 0.084 | Jirimutu et al. 2012 |
| Minke whale (*Balaenoptera acutorostrata*) | 0.086 | Yim et al. Nat. 2014 |
| Finless porpoise (*Neophocaena phocaenoides*) | 0.086 | Yim et al. Nat. 2014 |
| Koala (*Phascolarctos cinereus*) | 0.087 | Johnson et al. 2018 |
| Tibetan antelope (*Pantholops hodgsonii*) | 0.088 | Ge et al. Nat. 2013 |
| Yak(*Bos grunniens*) | 0.089 | Qiu et al. Nat. 2012 |
| Mongolian horse (*Equus ferus caballus*) | 0.089 | Huang et al. 2014 |
| Domestic bactrian camel (*Camelus bactrianus*) | 0.090 | Jirimutu et al. 2012 |
| South white Rhino (*Ceratotherium simum simum*) | 0.090 | Tunstall et al. 2018 |
| Common chimpanzee (*Pan troglodytes*) | 0.095 | Mikkelsen et al. 2005 |
| Domestic horse (*Equus caballus*) | 0.095 | Wade et al. 2009 |
| Cross River gorilla (*Gorilla gorilla diehli*) | 0.096 | Xue et al. 2015 |
| Wrangel wolly mammoth (*Mammuthus primigenius*) | 0.100 | Palkopoulou et al. 2016 |
| Polar bear (*Ursus maritimus*) | 0.108 | Liu et al. 2014 |
| Northern white Rhino (*Ceratotherium simum*) | 0.110 | Tunstall et al. 2018 |
| San Clemente Island fox (*Urocyon littoralis*) | 0.110 | Robinson et al. 2016 |
| Bactrian camel (*Camelus bactrianus*) | 0.116 | Wu et al. 2014 |
| Sumatran orangutan (*Pongo abelii*) | 0.120 | Locke et al. 2011 |
| Gray fox (*Urocyon cinereoargenteus*) | 0.120 | Robinson et al. 2016 |
| Brown hyena (*Parahyaena brunnea*) | 0.121 | Westbury et al. 2018 |
| Cow (*Bos taurus*) | 0.121 | Corbett-Detig et al. 2015 |
| Oimyakon wooly mammoth (*Mammuthus primigenius*) | 0.125 | Palkopoulou et al. 2016 |
| Rat (*Rattus norvegicus*) | 0.125 | Leffler et al. 2012 |
| Sumatran Rhinoceros (*Dicerorhinus sumatrensis*) | 0.130 | Mays et al. 2018 |
| Giant panda (*Ailuropoda melanoleuca*) | 0.132 | Li et al. 2010 |
| Siamang (*Symphalangus syndactylus*) | 0.130-0.150 | Carbone et al. 2014 |
| Bottlenose dolphin (*Tursiops truncatus*) | 0.142 | Yim et al. 2014 |
| Western lowland gorilla (*Gorilla gorilla gorilla*) | 0.144 | Xue et al. 2015 |
| Gray wolf (*Canis lupus*) | 0.149 | Corbett-Detig et al. 2015 |
| Fin whale (*Balaenoptera physalus*) | 0.151 | Yim et al. Nat. 2014 |
| Chinese hamster (*Cricetulus griseus*) | 0.159 | Lewis et al. 2013 |
| Silvery gibbon (*Hylobates moloch*) | 0.170 | Carbone et al. 2014 |
| Malaysian cynomolgus macaque (*Macaca fascicularis*) | 0.171 | Higashino et al. 2012 |
| Central African chimpanzees (*Pan troglodytes troglodytes*) | 0.176 | Mikkelsen et al. 2005 |
| Western lowland gorilla (*Gorilla gorilla gorilla*) | 0.178 | Scally et al. 2012 |
| Vervet monkey (*Chlorocebus aethiops pygerythrus*) | 0.180 | Warren et al. 2015 |
| Tibetan wild boars (*Sus scrofa*) | 0.182 | Li et al. 2013 |
| Olive baboon (*Papio anubis*) | 0.189 | Corbett-Detig et al. 2015 |
| San Rosa Island fox (*Urocyon littoralis*) | 0.191 | Robinson et al. 2016 |
| San Catalina Island fox (*Urocyon littoralis*) | 0.196 | Robinson et al. 2016 |
| San Cruz Island fox (*Urocyon littoralis*) | 0.197 | Robinson et al. 2016 |
| Northern white-cheeked gibbon (*Nomascus leucogenys*) | 0.220 | Carbone et al 2014 |
| Bighorn sheep (*Ovis canadensis*) | 0.222 | Corbett-Detig et al. 2015 |
| Alpaca (*Vicugna pacos*) | 0.266 | Wu et al. Nat. 2014 |
| David’s Myotis (*Myotis davidii*) | 0.279 | Zhang et al. 2013 |
| Rhesus macaque (*Macaca mulatta*) | 0.287 | Corbett-Detig et al. 2015 |
| Brown bear (*Ursus arctos*) | 0.320 | Liu et al. 2014 |
| Common marmoset (*Callithrix jacchus*) | 0.341 | Worley et al. 2014 |
| Przewalski's horse (*Equus ferus przewalskii*) | 0.363 | Corbett-Detig et al. 2015 |
| Brandt’s bat (*Myotis brandtii*) | 0.371 | Seim et al. 2013 |
| Chinese rhesus macaque (*Macaca mulatta lasiota*) | 0.410 | Yan et al. 2011 |
| Wild boar (*Sus scrofa*) | 0.441 | Corbett-Detig et al. 2015 |
| Black flying fox (*Pteropus alecto*) | 0.453 | Zhang et al. 2013 |
| Opossum (*Monodelphis domestica*) | 0.490 | Mikkelsen et al. 2007 |
| Crab-eating macaque (*Macaca fascicularis*) | 0.530 | Yan et al. 2011 |
| Rabbit (*Oryctolagus cuniculus*) | 0.750 | Carneiro et al. 2014 |
| Eastern hoolock gibbon (*Hoolock leuconedys*) | 0.800 | Carbone et al. 2014 |
| House mouse (*Mus musculus castaneus*) | 0.809 | Corbett-Detig et al. 2015 |

**Table S5 Maximum Likelihood values (MaxL) and AIC values for demographic models based on different numbers of bottleneck events for MJA, MJB, MPA, and MPB.**

**Table S6 Maximum Likelihood values (MaxL) and AIC values for demographic models based on further recent reduction events for MJA, MJB, MPA, and MPB.**

**Table S7 Maximum Likelihood values (MaxL) and AIC values for demographic models based on different divergence time possibilities for MJ (MJA and MJB) and MP (MPA and MPB).**

**Table S8 Estimations of effective population size under maximum likelihood method and their confidence interval (CI) by simulating 100 independent site frequency spectra conditional on the optimal demographic scenario.**

| Species | Population | | *Ne* | Confidence interval | Time (Kya) | Confidence interval |
| --- | --- | --- | --- | --- | --- | --- |
| MJ | MJA | JA1 | 102,294 | 35,708-256,340 | 300.424 | 295.124-389.223 |
|  |  | JA2 | 79,935 | 21,680-91,438 | 128.279 | 100.556-193.474 |
|  |  | JA3 | 105,870 | 59,530-148,366 | 84.387 | 45.815-94.406 |
|  |  | JA4 | 2,142 | 1,659-2,884 | 3.197 | 2.942-28.926 |
|  | MJB | JB1 | 183,270 | 55,797-269,514 | 300.424 | 295.124-389.223 |
|  |  | JB2 | 70,041 | 23,694-81,021 | 257.010 | 202.431-290.854 |
|  |  | JB3 | 76,793 | 39,540-139,241 | 177.847 | 127.072-192.613 |
|  |  | JB4 | 19,989 | 29,12-26,578 | 134.942 | 58.859-154.071 |
|  |  | JB5 | 58,414 | 52,898-91,858 | 31.839 | 21.26-39.792 |
|  |  | JB6 | 56,024 | 39,672-59,598 | 0.169 | 0.152-19.012 |
| MP | MPA | PA1 | 42,341 | 36,870-175,436 | 130.792 | 70.646-146.604 |
|  |  | PA2 | 12,064 | 503-21,638 | 38.766 | 28.984-69.657 |
|  |  | PA3 | 39,391 | 35,446-53,430 | 17.459 | 13.754-30.038 |
|  |  | PA4 | 35,661 | 20,212-37,196 | 6.439 | 0.169-8.691 |
|  |  | PA5 | 4,137 | 3,388-16,926 | 0.024 | 0.004-0.093 |
|  | MPB | PB1 | 110,353 | 32,712-26,2871 | 130.792 | 70.646-146.604 |
|  |  | PB2 | 2,911 | 2,396-17,391 | 15.248 | 14.603-61.2 |
|  |  | PB3 | 26,510 | 21,605-81,511 | 12.299 | 2.907-18.474 |
|  |  | PB4 | 697 | 402-17,793 | 0.054 | 0.048-6.442 |

**Table S9 Estimation of migration rate under maximum likelihood method and their confidence interval (CI) by simulating 100 independent site frequency spectra conditional on the optimal demographic scenario.**

| Species | Migrate Events | Probability^a^ | Nm^b^ | Migrate Events | Probability^a^ | Nm^b^ |
| --- | --- | --- | --- | --- | --- | --- |
| MJ | JA1 to JB1 | 5.51E-04 | 100.9678 | JB1 to JA1 | 3.17E-08 | 0.0032 |
|  | JA1 to JB2 | 5.62E-05 | 3.9392 | JB2 to JA1 | 4.60E-09 | 0.0005 |
|  | JA1 to JB3 | 2.58E-08 | 0.0020 | JB3 to JA1 | 2.86E-03 | 292.2744 |
|  | JA1 to JB4 | 1.76E-07 | 0.0135 | JB4 to JA1 | 9.25E-04 | 73.9719 |
|  | JA2 to JB4 | 1.39E-05 | 0.2786 | JB4 to JA2 | 1.23E-03 | 98.4400 |
|  | JA3 to JB4 | 2.65E-09 | 0.00005 | JB4 to JA3 | 6.07E-10 | 0.00006 |
|  | JA3 to JB5 | 3.23E-07 | 0.0189 | JB5 to JA3 | 2.75E-05 | 2.9100 |
|  | JA4 to JB5 | 1.29E-05 | 0.7532 | JB5 to JA4 | 1.29E-05 | 0.0276 |
|  | JA4 to JB6 | 3.74E-05 | 2.0940 | JB6 to JA4 | 1.12E-05 | 0.0239 |
| MP | PA1 to PB1 | 3.12E-04 | 34.4725 | PB1 to PA1 | 2.78E-09 | 0.0001 |
|  | PA2 to PB1 | 8.37E-06 | 0.9239 | PB1 to PA2 | 7.62E-09 | 0.0001 |
|  | PA3 to PB1 | 1.01E-09 | 0.0001 | PB1 to PA3 | 1.31E-05 | 0.5158 |
|  | PA3 to PB2 | 1.92E-08 | 0.0001 | PB2 to PA3 | 3.30E-07 | 0.0130 |
|  | PA3 to PB3 | 5.80E-09 | 0.0002 | PB3 to PA3 | 1.81E-09 | 0.0001 |
|  | PA4 to PB3 | 1.36E-06 | 0.0362 | PB3 to PA4 | 6.19E-07 | 0.0221 |
|  | PA4 to PB4 | 2.49E-07 | 0.0002 | PB4 to PA4 | 1.85E-06 | 0.0658 |
|  | PA5 to PB4 | 3.92E-04 | 0.2734 | PB4 to PA5 | 1.24E-04 | 0.5144 |

^a^ The probability of the source population to the target population.

^b^ Nm represents the number of immigrant per generation.

**Table S10 The genetic diversity comparison between simulation data sets, which are conditional on the inferred final demographic scenario, and observed data set.**

| Population | Sample size | K_observed_ | K_simulated_ | | Difference (%) | $\theta$_observed_ (‰) | $\theta$_simulated_ (‰) | $\pi$_observed_ (‰) | $\pi$_simulated_ (‰) |
| --- | --- | --- | --- | --- | --- | --- | --- | --- | --- |
| MJA | 82 | 2,895,275 | | 3,303,181 | 14.089 | 0.238 | 0.272 | 0.274 | 0.350 |
| MJB | 64 | 16,453,989 | | 16,719,134 | 1.611 | 1.426 | 1.450 | 1.128 | 1.153 |
| MPA | 28 | 9,970,736 | 10,057,186 | | 0.867 | 1.050 | 1.059 | 0.920 | 0.940 |
| MPB | 18 | 7,638,420 | 7,727,367 | | 1.164 | 0.910 | 0.921 | 0.817 | 0.843 |

**Table S11 GO and KEGG enrichment analyses of genes affected by homozygous loss-of-function (LOF) mutations.**

| Population | Category | Gene Count | p-Value | List Total |
| --- | --- | --- | --- | --- |
| MJA  (232genes) | KEGG:hsa04512:ECM-receptor interaction | 4 | 4.89E-02 | 76 |
|  | KEGG:hsa05134:Legionellosis | 4 | 2.07E-02 | 76 |
|  | KEGG:hsa05144:Malaria | 3 | 4.94E-02 | 76 |
|  | KEGG:hsa04640:Hematopoietic cell lineage | 5 | 1.47E-02 | 76 |
|  | GO:0002040~sprouting angiogenesis | 3 | 3.38E-02 | 196 |
|  | GO:1900748~positive regulation of vascular endothelial growth factor signaling pathway | 2 | 3.44E-02 | 196 |
|  | GO:0016525~negative regulation of angiogenesis | 4 | 3.54E-02 | 196 |
|  | GO:0042632~cholesterol homeostasis | 4 | 3.83E-02 | 196 |
|  | GO:0051142~positive regulation of NK T cell proliferation | 2 | 4.57E-02 | 196 |
|  | GO:0007169~transmembrane receptor protein tyrosine kinase signaling pathway | 7 | 8.85E-04 | 196 |
|  | GO:0007010~cytoskeleton organization | 7 | 1.13E-02 | 196 |
|  | GO:0045041~protein import into mitochondrial intermembrane space | 2 | 2.31E-02 | 196 |
|  | GO:0010842~retina layer formation | 3 | 2.66E-02 | 196 |
|  | GO:0072659~protein localization to plasma membrane | 4 | 3.68E-02 | 196 |
|  | GO:0014816~skeletal muscle satellite cell differentiation | 2 | 4.57E-02 | 196 |
|  | GO:0006812~cation transport | 3 | 4.72E-02 | 196 |
|  | GO:0031647~regulation of protein stability | 4 | 4.79E-02 | 196 |
|  | GO:0016020~membrane | 38 | 3.72E-03 | 197 |
|  | GO:0005813~centrosome | 12 | 6.57E-03 | 197 |
|  | GO:0031012~extracellular matrix | 9 | 1.50E-02 | 197 |
|  | GO:0031430~M band | 3 | 2.51E-02 | 197 |
|  | GO:1902495~transmembrane transporter complex | 2 | 3.19E-02 | 197 |
|  | GO:0030018~Z disc | 5 | 3.86E-02 | 197 |
|  | GO:0016323~basolateral plasma membrane | 6 | 4.53E-02 | 197 |
|  | GO:0043235~receptor complex | 5 | 4.84E-02 | 197 |
|  | GO:0005509~calcium ion binding | 18 | 3.26E-03 | 192 |
|  | GO:0019899~enzyme binding | 11 | 4.73E-03 | 192 |
|  | GO:0002020~protease binding | 6 | 5.81E-03 | 192 |
|  | GO:0030246~carbohydrate binding | 7 | 2.42E-02 | 192 |
|  | GO:0005249~voltage-gated potassium channel activity | 4 | 2.79E-02 | 192 |
|  | GO:0004222~metalloendopeptidase activity | 5 | 3.95E-02 | 192 |
| MPP  (305genes) | KEGG:hsa04512:ECM-receptor interaction | 7 | 8.63E-04 | 90 |
|  | KEGG:hsa05146:Amoebiasis | 6 | 1.18E-02 | 90 |
|  | KEGG:hsa05200:Pathways in cancer | 12 | 1.20E-02 | 90 |
|  | KEGG:hsa05205:Proteoglycans in cancer | 7 | 4.44E-02 | 90 |
|  | GO:0001525~angiogenesis | 9 | 2.26E-02 | 260 |
|  | GO:0030949~positive regulation of vascular endothelial growth factor receptor signaling pathway | 3 | 2.47E-02 | 260 |
|  | KEGG:hsa04015:Rap1 signaling pathway | 9 | 5.59E-03 | 90 |
|  | KEGG:hsa04611:Platelet activation | 7 | 6.55E-03 | 90 |
|  | KEGG:hsa04510:Focal adhesion | 12 | 6.27E-05 | 90 |
|  | KEGG:hsa04151:PI3K-Akt signaling pathway | 10 | 3.36E-02 | 90 |
|  | KEGG:hsa04730:Long-term depression | 4 | 4.22E-02 | 90 |
|  | GO:0030154~cell differentiation | 14 | 2.75E-02 | 260 |
|  | GO:0051301~cell division | 6 | 4.55E-02 | 260 |
|  | GO:0072659~protein localization to plasma membrane | 5 | 2.85E-03 | 260 |
|  | GO:0008277~regulation of G-protein coupled receptor protein signaling pathway | 4 | 2.98E-03 | 260 |
|  | GO:0045663~positive regulation of myoblast differentiation | 3 | 3.93E-03 | 260 |
|  | GO:0043931~ossification involved in bone maturation | 5 | 4.73E-03 | 260 |
|  | GO:0021987~cerebral cortex development | 9 | 1.08E-02 | 260 |
|  | GO:0030198~extracellular matrix organization | 4 | 1.12E-02 | 260 |
|  | GO:0070830~bicellular tight junction assembly | 4 | 1.29E-02 | 260 |
|  | GO:0009968~negative regulation of signal transduction | 3 | 1.53E-02 | 260 |
|  | GO:0090303~positive regulation of wound healing | 3 | 1.91E-02 | 260 |
|  | GO:0048009~insulin-like growth factor receptor signaling pathway | 4 | 1.91E-02 | 260 |
|  | GO:0032467~positive regulation of cytokinesis | 4 | 1.92E-02 | 260 |
|  | GO:0034446~substrate adhesion-dependent cell spreading | 5 | 2.06E-02 | 260 |
|  | GO:0016485~protein processing | 14 | 2.51E-02 | 260 |
|  | GO:0007411~axon guidance | 3 | 3.69E-02 | 260 |
|  | GO:0006541~glutamine metabolic process | 3 | 3.75E-02 | 260 |
|  | GO:0007130~synaptonemal complex assembly | 4 | 4.10E-02 | 260 |
|  | GO:0007157~heterophilic cell-cell adhesion via plasma membrane cell adhesion molecules | 11 | 4.18E-02 | 260 |
|  | GO:0035583~sequestering of TGFbeta in extracellular matrix | 2 | 4.56E-02 | 260 |
|  | GO:0072137~condensed mesenchymal cell proliferation | 2 | 4.56E-02 | 260 |
|  | GO:0031012~extracellular matrix | 18 | 1.45E-06 | 263 |
|  | GO:0005604~basement membrane | 7 | 9.64E-04 | 263 |
|  | GO:0005929~cilium | 8 | 6.27E-03 | 263 |
|  | GO:0005886~plasma membrane | 77 | 9.08E-03 | 263 |
|  | GO:0007155~cell adhesion | 19 | 2.90E-04 | 260 |
|  | GO:0030658~transport vesicle membrane | 4 | 1.71E-02 | 263 |
|  | GO:0005814~centriole | 6 | 2.37E-02 | 263 |
|  | GO:0043235~receptor complex | 6 | 3.66E-02 | 263 |
|  | GO:0031982~vesicle | 6 | 3.76E-02 | 263 |
|  | GO:0005911~cell-cell junction | 7 | 3.83E-02 | 263 |
|  | GO:0030286~dynein complex | 3 | 3.94E-02 | 263 |
|  | GO:0005578~proteinaceous extracellular matrix | 9 | 4.02E-02 | 263 |
|  | GO:0005606~laminin-1 complex | 2 | 4.25E-02 | 263 |
|  | GO:0005813~centrosome | 12 | 4.51E-02 | 263 |
|  | GO:0000795~synaptonemal complex | 3 | 4.62E-02 | 263 |
|  | GO:0005509~calcium ion binding | 24 | 3.63E-04 | 248 |
|  | GO:0005178~integrin binding | 7 | 4.45E-03 | 248 |
|  | GO:0005515~protein binding | 147 | 1.46E-02 | 248 |
|  | GO:0002020~protease binding | 6 | 1.64E-02 | 248 |
|  | GO:0004714~transmembrane receptor protein tyrosine kinase activity | 4 | 1.79E-02 | 248 |
|  | GO:0005524~ATP binding | 32 | 3.03E-02 | 248 |
|  | GO:0004871~signal transducer activity | 8 | 3.08E-02 | 248 |
|  | GO:0044822~poly(A) RNA binding | 25 | 4.21E-02 | 248 |
|  | GO:0004175~endopeptidase activity | 4 | 4.45E-02 | 248 |

The ECM-receptor interaction, cancer /disease and angiogenesis pathway that were enriched in both MJA and MPP populations are highlighted in red, orange and yellow, respectively. Additionally, the cholesterol homeostasis which enriched in MJA and highlighted in blue.

**
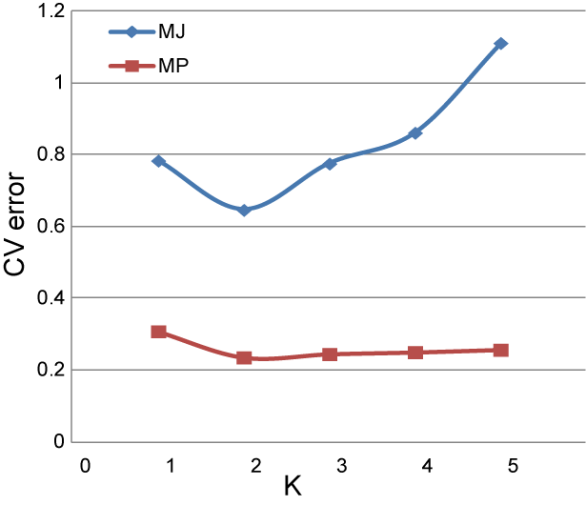
**

**Figure S1 Changes in cross-validation (CV) error in the admixture analyses of MJ and MP when the postulated number of ancestral clusters (K) was set from 2 to 5 in ADMIXTURE analyses.**

**
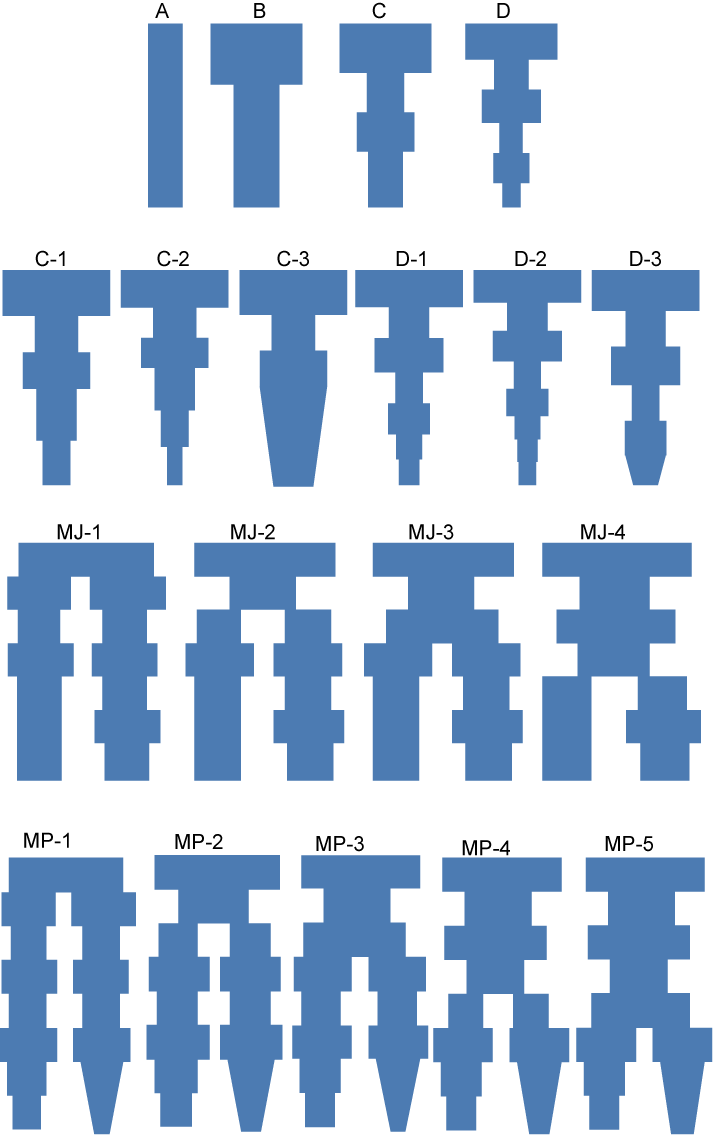
**

**Figure S2 Demographic history reconstruction by simulating a series of increasingly complex demographic models.** First, we established four demographic models based on different numbers of bottleneck events (Model-A to Model-D). The AIC results identify the preferred model for MJA with two bottlenecks (Model C), and for MJB, MPA, and MPB a model with three bottlenecks (Model D). Based on the preferred models for the four groups (Model C and Model D), we then explored whether they have experienced further recent reduction events, considering the catastrophic effects of illegal trading and anthropogenic disturbances on population sizes of these two pangolins. Model-C-1 and Model-D-1 add one recent reduction, and Mode-C-2 and Model-D-2 add two. Model-C-3 and Model-D-3 add exponential reduction. The AIC results manifest that for MJA and MJB, Model C and Model D remain the preferred demographic scenarios, respectively, suggesting no further recent reduction event. For MPA and MPB, Model-D-1 and Model-D-3, which add one recent reduction and exponential reduction, were preferred, respectively. We merged the preferred demographic scenarios to form the optimal model for the demographic history of MJ (Model C for MJA and Model D for MJB) and MP (Model-D-1 for MPA and Model-D-3 for MPB). While fixing the merged demographic models of MJ and MP as described above, we further incorporate different divergence time possibilities in the models to clarify the divergence time of the two groups in MJ (MJA and MJB) and MP (MPA and MPB). Four models for MJ (Model-MJ-1 to Model-MJ-4) and five models for MP (Model-MP-1 to Model-MP-5) were compared. The results prefer Model-MJ-1, in which MJA and MJB diverge from their most recent common ancestor (MRCA) and Model-MP-3, in which MPA and MPB diverge after the first bottleneck, as the best models for expounding the demographic history of MJ and MP.

**
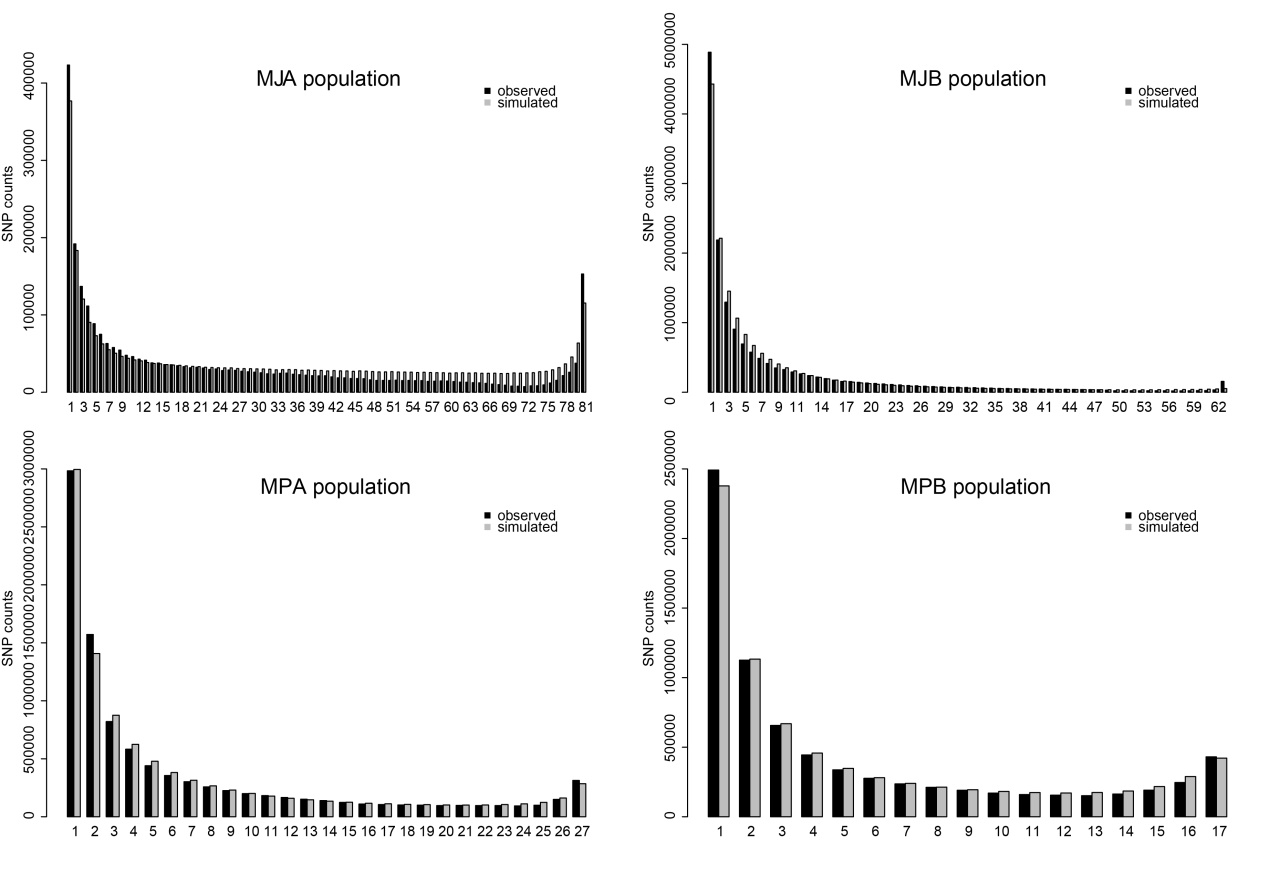
**

**Figure S3 Site frequency spectrum comparison between simulated and observed data sets for MJA, MJB, MPA, and MPB populations.**

**
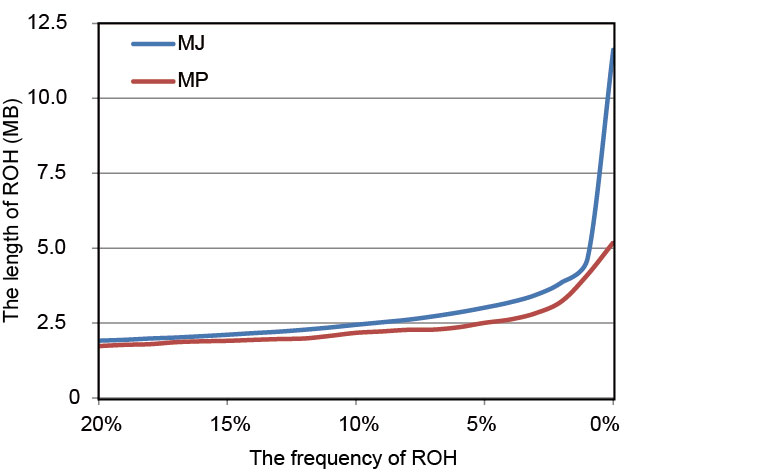
**

**Figure S4 Difference of ROH frequency between MJ and MP.**

**References**

Abascal F, Corvelo A and Cruz F et al. Extreme genomic erosion after recurrent demographic bottlenecks in the highly endangered Iberian lynx. *Genome Biol* 2016; **17**: 251.

Carbone L, Harris RA and Gnerre S et al. Gibbon genome and the fast karyotype evolution of small apes. *Nature* 2014; **513**: 195-201.

Carneiro M, Rubin CJ and Di Palma F et al. Rabbit genome analysis reveals a polygenic basis for phenotypic change during domestication. *Science* 2014; **345**: 1074-9.

Cho YS, Hu L and Hou H et al. The tiger genome and comparative analysis with lion and snow leopard genomes. *Nature Com* 2013; **4**: 2433.

Choo SW, Rayko M and Tan TK et al. Pangolin genomes and the evolution of mammalian scales and immunity. *Genome Res* 2016; **26**: 1312-22.

Corbett-Detig RB, Hartl DL and Sackton TB et al. Natural selection constrains neutral diversity across a wide range of species. *PLoS Biol* 2015; **13**: e1002112.

Dalloul RA, Long JA and Zimin AV et al. Multi-platform next-generation sequencing of the domestic turkey (*Meleagris gallopavo*): genome assembly and analysis. *PLoS Biol* 2010; **8**: e1000475.

Dobrynin P, Liu S and Tamazian G et al. Genomic legacy of the African cheetah, *Acinonyx jubatus*. *Genome Biol* 2015; **16**: 277.

Ge RL, Cai Q and Shen YY et al. Draft genome sequence of the Tibetan antelope. *Nat Commun* 2013; **4**: 1858.

Higashino A, Sakate R and Kameoka Y et al. Whole-genome sequencing and analysis of the Malaysian cynomolgus macaque (*Macaca fascicularis*) genome. *Genome Biol* 2012; **13**: R58.

Huang J, Zhao Y and Shiraigol W et al. Analysis of horse genomes provides insight into the diversification and adaptive evolution of karyotype. *Sci Rep* 2014; **4**: 4958.

Huang Z, Xu J and Xiao S et al. Comparative optical genome analysis of two pangolin species: *Manis pentadactyla* and *Manis javanica*. *Gigascience* 2016; **5**: 1-5.

Jirimutu, Wang Z and Ding G et al. Genome sequences of wild and domestic bactrian camels. *Nat Commun* 2012; **3**: 1202.

Johnson RN, O'Meally D and Chen Z et al. Adaptation and conservation insights from the koala genome. *Nat Genet* 2018; **50**: 1102-11.

[Kim EB](https://www.ncbi.nlm.nih.gov/pubmed/?term=Kim%20EB%5BAuthor%5D&cauthor=true&cauthor_uid=21993625), [Fang X](https://www.ncbi.nlm.nih.gov/pubmed/?term=Fang%20X%5BAuthor%5D&cauthor=true&cauthor_uid=21993625) and [Fushan AA](https://www.ncbi.nlm.nih.gov/pubmed/?term=Fushan%20AA%5BAuthor%5D&cauthor=true&cauthor_uid=21993625) et al. Genome sequencing reveals insights into physiology and longevity of the naked mole rat. 2011; **479**: 223-7.

[Leffler EM](https://www.ncbi.nlm.nih.gov/pubmed/?term=Leffler%20EM%5BAuthor%5D&cauthor=true&cauthor_uid=22984349), [Bullaughey K](https://www.ncbi.nlm.nih.gov/pubmed/?term=Bullaughey%20K%5BAuthor%5D&cauthor=true&cauthor_uid=22984349) and [Matute DR](https://www.ncbi.nlm.nih.gov/pubmed/?term=Matute%20DR%5BAuthor%5D&cauthor=true&cauthor_uid=22984349) et al. Revisiting an old riddle: What determines genetic diversity levels within species? *PLoS Biol* 2012; **10**: e1001388.

Lewis NE, Liu X and Li Y et al. Genomic landscapes of Chinese hamster ovary cell lines as revealed by the Cricetulus griseus draft genome. *Nat Biotechnol* 2013; **31**: 759-765.

Li M, Tian S and Jin L et al. Genomic analyses identify distinct patterns of selection in domesticated pigs and Tibetan wild boars. *Nat Genet* 2013; **45**: 1431-81438.

Li R, Fan W and Tian G et al. The sequence and de novo assembly of the giant panda genome. *Nature* 2010; **463**: 311-7.

Lindblad-Toh K, Wade CM and Mikkelsen TS et al. Genome sequence, comparative analysis and haplotype structure of the domestic dog. *Nature* 2005; **438**: 803-19.

Liu S, Lorenzen ED and Fumagalli M et al. Population genomics reveal recent speciation and rapid evolutionary adaptation in polar bears. *Cell* 2014; **2157**: 785-94.

Locke DP, Hillier LW and Warren WC et al. Comparative and demographic analysis of orangutan genomes. *Nature* 2011; **469**: 529-33.

Mays J, Herman L and Hung CM et al. Genomic analysis of demographic history and ecological niche modeling in the endangered sumatran rhinoceros *Dicerorhinus sumatrensis*. *Curr Biol* 2018; **28**:70-6.

Meyer M, Kircher M and Gansauge MT et al. A high-coverage genome sequence from an archaic Denisovan individual. *Science* 2012; **338**: 222-6.

Mikkelsen TS, Hillier LDW and Eichler EE et al. Initial sequence of the chimpanzee genome and comparison with the human genome. *Nature* 2005; **437**: 69-87.

Mikkelsen TS, Wakefield MJ and Aken B et al. Genome of the marsupial Monodelphis domestica reveals innovation in non-coding sequences. *Nature* 2007; **447**:167-77.

Palkopoulou E, Mallick S and Skoglund P et al. Complete genomes reveal signatures of demographic and genetic declines in the woolly mammoth. *Curr Biol* 2015; **25**:1395-400.

Perry GH, Reeves D and Melsted P et al. A genome sequence resource for the aye-aye (*Daubentonia madagascariensis*), a nocturnal lemur from Madagascar. *Genome Biol Evol* 2012; **4**: 126-35.

Prüfer K, Racimo F and Patterson N et al. The complete genome sequence of a Neanderthal from the Altai Mountains. *Nature* 2014; **505**: 43-9.

Qiu Q, Zhang G and Ma T et al. The yak genome and adaptation to life at high altitude. *Nat Genet* 2012; **44**: 946-94.

Robinson J, Ortega-Del Vecchyo D and Fan Z et al. Genomic flatlining in the endangered island fox. *Curr Biol* 2016; **26**: 1183-9.

Scally A, Dutheil JY and Hillier LW et al. Insights into hominid evolution from the gorilla genome sequence. *Nature* 2012; **483**: 169-75.

Seim I, Fang X and Xiong Z et al. Genome analysis reveals insights into physiology and longevity of the Brandt's bat *Myotis brandtii*. *Nat Commun* 2013; **4**: 2212.

Tunstall T, Kock R and Vahala J et al. Evaluating recovery potential of the northern white rhinoceros from cryopreserved somatic cells. *Genome Res* 2018; **28**:780-8.

Wade CM, Giulotto E and Sigurdsson S et al. Genome sequence, comparative analysis, and population genetics of the domestic horse. *Science* 2009; **326**: 865-7.

Warren WC, Jasinska AJ and García-Pérez R et al. The genome of the vervet (*Chlorocebus aethiops sabaeus*). *Genome Res* 2015; **25**: 1921-33.

Westbury MV, Hartmann S and Barlow A et al. Extended and continuous decline in effective population size results in low genomic diversity in the world's rarest hyena species, the brown hyena. *Mol Biol Evol* 2018; **35**:1225-37

Worley KC, Warren WC and Rogers J et al. The common marmoset genome provides insight into primate biology and evolution. *Nat Genet* 2014; **46**: 850-7.

Wu H, Guang X and Al-Fageeh MB et al. Camelid genomes reveal evolution and adaptation to desert environments. *Nat Commun* 2014; **5**: 5188.

Xue Y, Prado Martinez J and Sudmant PH et al. Mountain gorilla genomes reveal the impact of long-term population decline and inbreeding. *Science* 2015; **348**: 242-5.

Yan G, Zhang G and Fang X et al. Genome sequencing and comparison of two nonhuman primate animal models, the cynomolgus and Chinese rhesus macaques. *Nat Biotechnol* 2011; **29**: 1019-23.

Yim HS, Cho YS and Guang X et al. Minke whale genome and aquatic adaptation in cetaceans. *Nat Genet* 2014; **46**: 88-92.

Zhang G, Cowled C and Shi Z et al. Comparative analysis of bat genomes provides insight into the evolution of flight and immunity. *Science* 2013; **339**: 456-60.

Zhou X, Meng X and Liu Z et al. Population genomics reveals low genetic diversity and adaptation to hypoxia in snub-nosed monkeys. *Mol Biol Evol* 2016 **33**: 2670-81.

Zhou X, Sun F and Xu S et al. Baiji genomes reveal low genetic variability and new insights into secondary aquatic adaptations. *Nat Commun* 2013; **4**: 2708.
